# Supplementary material for: Isolation, Purification, and Characterisation of a Phage Tail-Like Bacteriocin from the Insect Pathogenic Bacterium Brevibacillus laterosporus
Source: Biomolecules. 2022 Aug 20;12(8):1154. doi: 10.3390/biom12081154 (PMC9406221; doi:10.3390/biom12081154)

Supplementary Material (SM)

Table S1. Spectrophotometer reading (OD_{600nm}) of *Bl* 1821L culture after treatment with the mitomycin C at various concentrations.

Treatments	Time intervals (Hours)					% Decrease in OD_{600nm}
	0	2	4	6	24	
Mitomycin C (1 µg/mL)	1.41	1.44	1.46	1.48	0.71	49.65
Mitomycin C (3 µg/mL)	1.56	1.56	1.57	1.58	0.92	41.03
Control	1.30	1.34	1.47	1.49	1.35	-3.85
*LSD (5%) (Control vs Treated)	0.18	0.15	0.12	0.12	0.37	24.49
LSD (5%) (**MMC1 vs ***MMC3)	0.16	0.14	0.12	0.11	0.34	22.68

Note: *LSD= Least significant digit

**MMC1= Mitomycin C @ 1 µg/mL

***MMC3= Mitomycin C @ 3 µg/mL

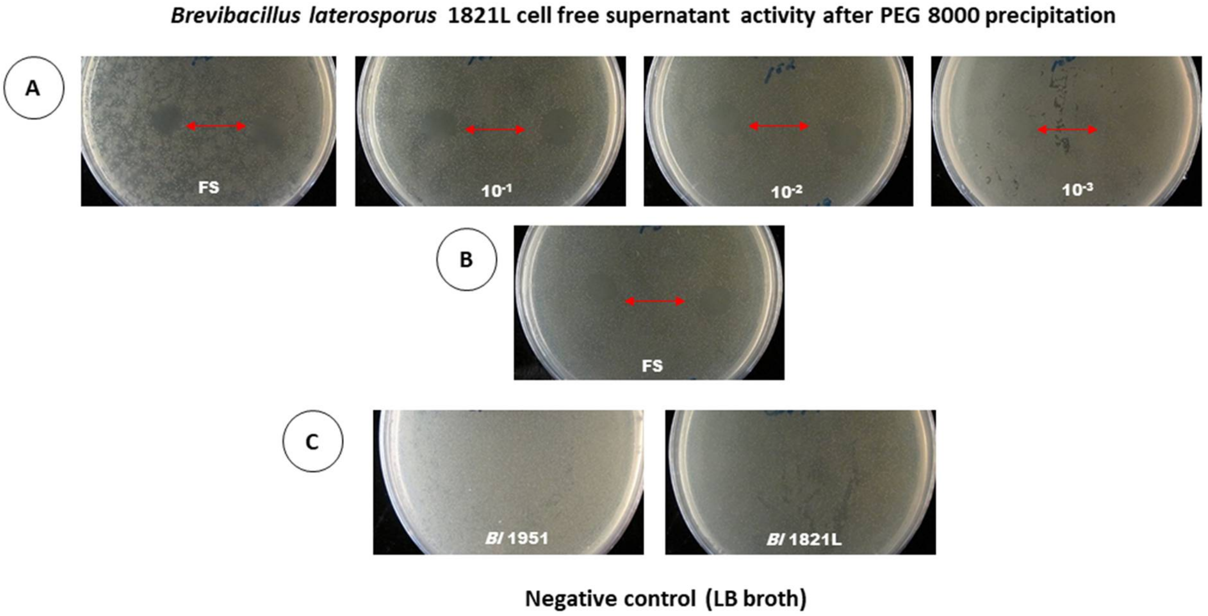

Figure S1. **Antibacterial activity of *Bl* 1821L cell free supernatant after PEG 8000 precipitation in the serial dilutions assay test against *Bl* 1951 as the host bacterium (A) and *Bl* 1821L as the host bacterium (B). Arrows (red colour) denote the zones of inhibition due to the activity of PEG 8000 precipitated putative antibacterial proteins. LB broth was used as a negative control against both the strains (*Bl* 1951 & *Bl* 1821L) (C).**

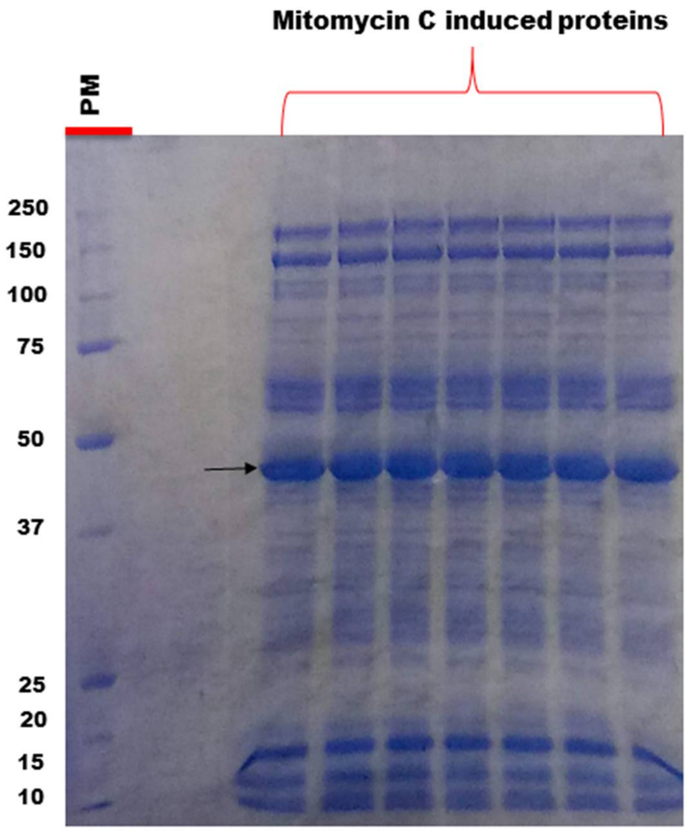

Figure S2. SDS-PAGE of mitomycin C induced culture of *Bl* 1821L showing a prominent band of ~48 kD (shown with dark arrow).

Note: Based on its prominence, ~48 kD protein band was hypothesised to be involved in the putative antibacterial activity. Therefore, the band was excised for preliminary N-terminal sequencing and the resultant short sequence amino acids revealed several hits (covering 70% of the amino acid sequence) to the loci A0A518VEB0 in the *Bl* 1821L genome (NZ_CP033464.1) encoding a predicted defective phage protein, similar to that encoded by the *Bs* 168 phage-like element PBSX gene *xkdK*. Furthermore, these findings were authenticated with the subsequent TEM examination, SDS-PAGE, N-terminal sequencing, and bioinformatic analysis of purified ~48 kD protein.

Refer to N-terminal sequencing of ~48 kD crude protein:

(https://data.lincoln.ac.nz/ndownloader/files/30948223)

***Brevibacillus laterosporus* 1821L induced cultures after PEG 8000 precipitation**

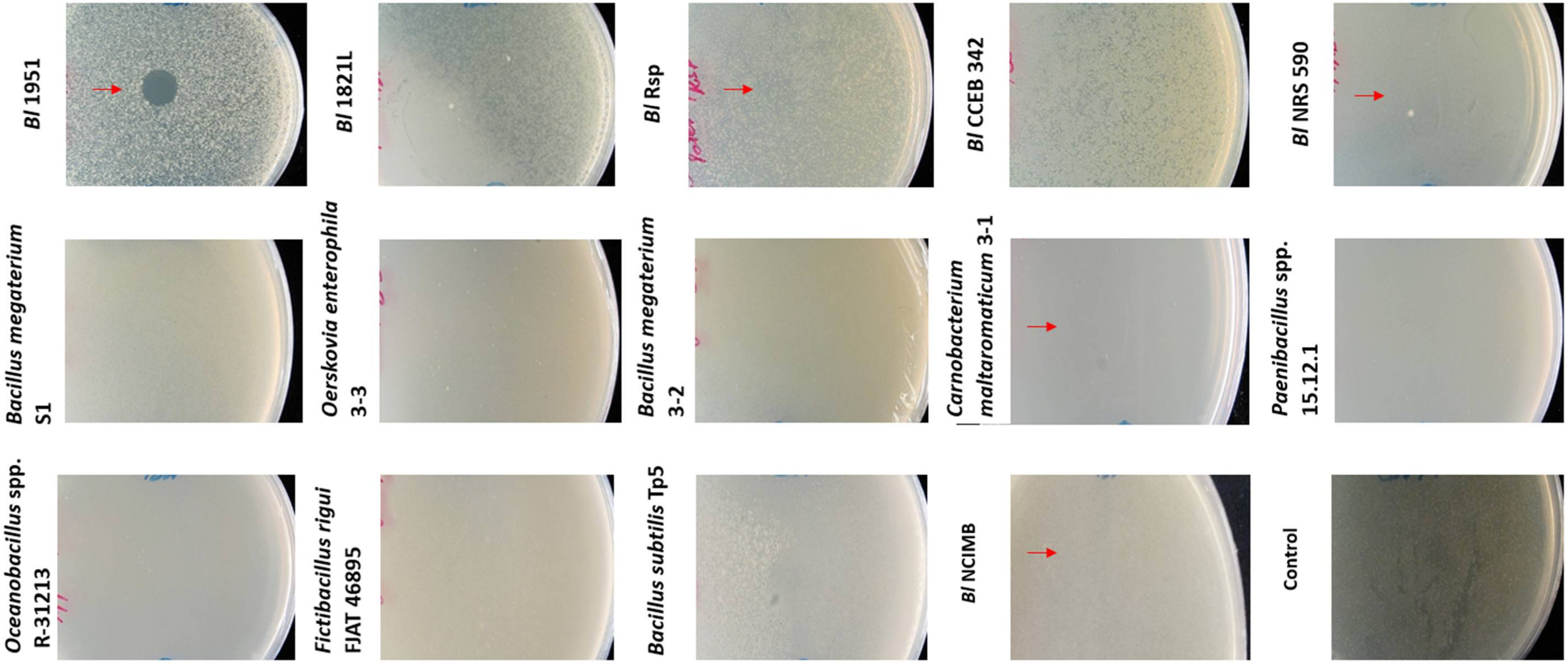

Figure S3. Antibacterial activity of *Bl* 1821L induced culture cell free supernatant after PEG 8000 precipitation against various gram-positive bacteria. Arrows (red colour) denote the zone of inhibition due to the activity of PEG 8000 precipitated putative antibacterial proteins.

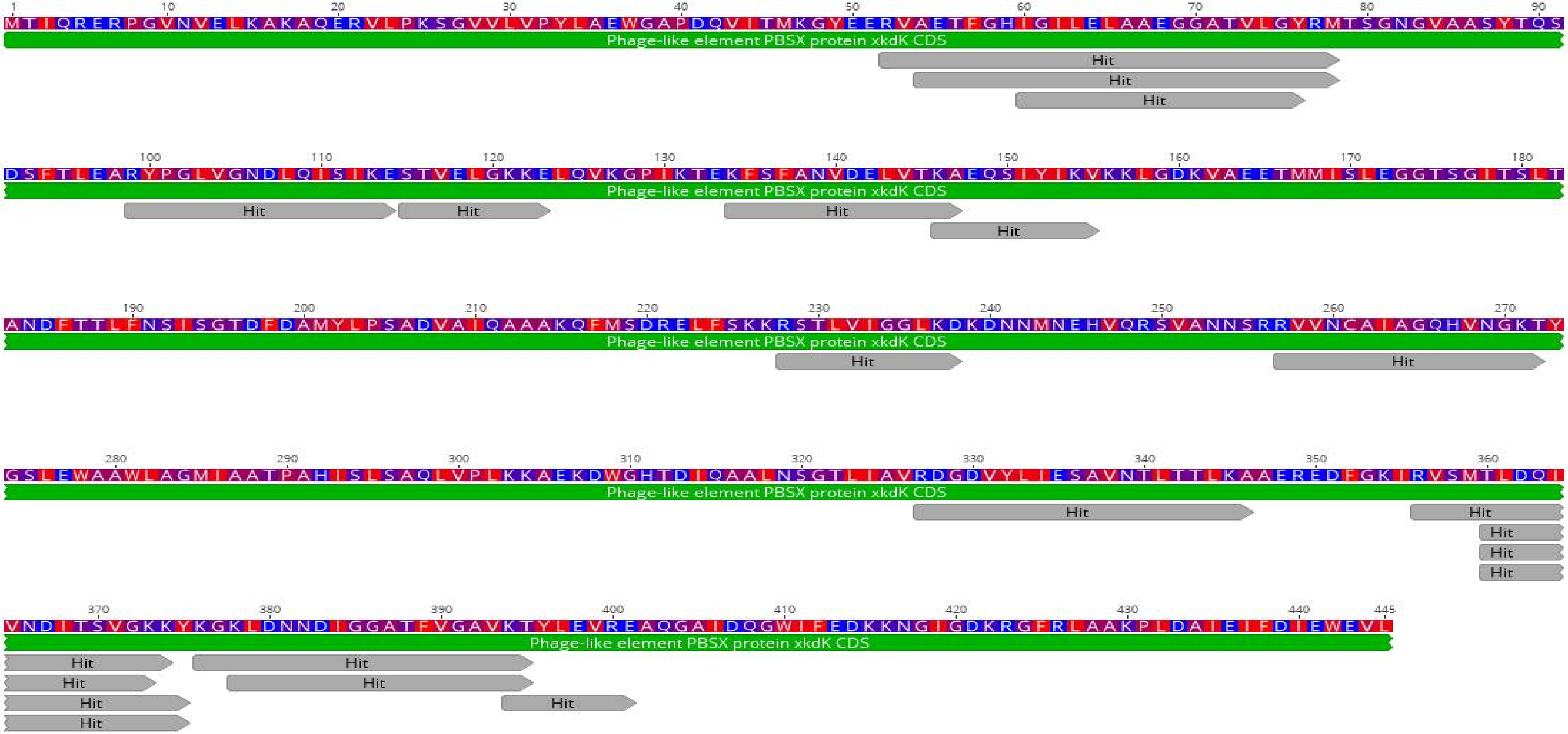

Figure S4. N-terminal sequenced short amino acid match hits to the putative phage tail protein (~48 kD) encoded in *Bl* 1821L genome.

Refer to N-terminal sequencing of ~48 kD purified protein: **(https://data.lincoln.ac.nz/ndownloader/files/30948616)**

Table S2. Similar proteins to the ~48 kD identified putative phage tail protein of *Bl* 1821L in the Uniprot database.

Similar proteins	Organism	Gene name	Length
Phage tail protein	*Brevibacillus laterosporus* (*Bacillus laterosporus*)	C4A76_07870, C4A77_13935, D5F52_00915	445
Phage tail protein	*Brevibacillus laterosporus* (*Bacillus laterosporus*)	EX87_06735	445
Phage tail-sheath protein	*Brevibacillus laterosporus* LMG 15441	BRLA_c036460	462
Uncharacterised protein	*Brevibacillus borstelensis* GI-9	BLGI_826	445
Phage tail protein	*Brevibacillus laterosporus* (*Bacillus laterosporus*)	EEL32_11960	445
Phage tail protein	*Brevibacillus laterosporus* SKDU 10	AYJO8_14030	445
Phage tail protein	*Brevibacillus laterosporus* (*Bacillus laterosporus*)	EX87_02320	445
Phage tail protein	*Brevibacillus laterosporus* (*Bacillus laterosporus*)	D5F52_16160	445
Phage tail protein	*Brevibacillus laterosporus* (*Bacillus laterosporus*)	C4A76_21720	445

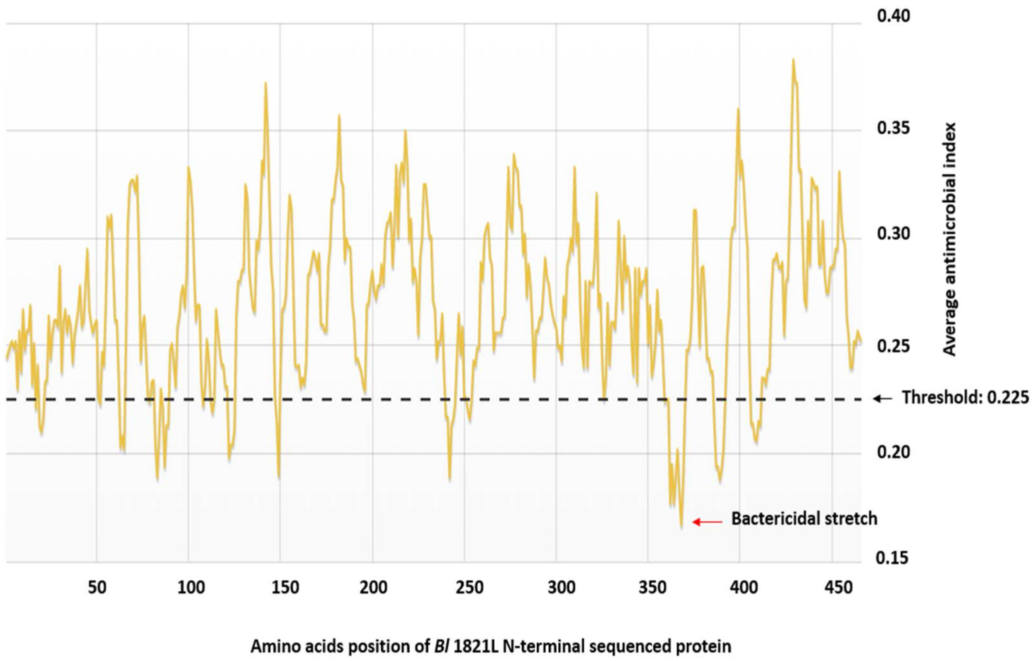

Figure S5. AMPA analysis of identified phage tail-sheath protein of *Bl* 1821L. Identified bactericidal motif is indicated is pointed with red arrow.

**1**	MNGGTFTTGKEKERAGIYFNFKTTAQERVSLSERGTVALPVASSWGEAKTFVSISSVEDL
**61**	NKKVGLSIDDPSLLLLREAKKNAKTVLMYRLTEGVRASADIAEGVKATAVYGGTKGNDII
**121**	IRINQNVLDANSFDVTTYMDESEVDKQTVKKAEELTANGYVTFTGTGDLSSTIPLTGSEG
**181**	DTAAETLNASAGIRLSGGTDKAPVNSDYTDFLAAAETESFDVIALPVAEGDQLKATFAAF
**241**	IKRLRDGQGQKVQGVTANYAGDYEGIINVTEGVLLEDGTEVTPDKATAWVAGASAGATN
**301**	QSLTFVEYEGAVDVLHRLDHDTIVERLGKGEFLFTFDARDKSVSVEKDINSLVTFTAEKN
**361**	KKFAKNKIVRVLDAVNNDLTRELKALIKSRKGSGSDIPASEDGLQYVKTMITQYMTTLQD
**421**	AGGITGFDSDEDITISMNEDRDGFLIDLAVQPVDAAEKFYFNVEVN

Figure S6. AMPA analysis of identified phage-like element PBSX protein XkdK of *Bl* 1821L. Amino acids (360-373) corresponding to the bactericidal stretch are highlighted in red colour.

Table S3. Effect of crude *Bl* 1821L putative antibacterial proteins (ABPs) on the number of viable cells of *Bl* 1821L and *Bl* 1951 after incubation at 30°C for various time intervals. Data presents the mean values of four experiments. Values of % decrease/increase in the number of viable cells are calculated from CFUs values of corresponding time intervals.

Time intervals (Hours)	*Bl* 1821L	*Bl* 1821L + *Bl* 1821L crude ABPs	% Decrease/increase in no. of viable cells	*Bl* 1951	*Bl* 1951 + *Bl* 1821L crude ABPs	% Decrease/increase in no. of viable cells	*LSD (5%)
1	4.46E+06 (6.650)**	5.56E+06 (6.745)	-24.65	3.18E+06 (6.502)	4.19E+06 (6.622)	-31.89	0.350
3	3.70E+06 (6.568)	3.85E+06 (6.585)	-4.05	3.64E+06 (6.561)	4.93E+06 (6.692)	-35.40	0.281
6	3.58E+06 (6.553)	2.50E+06 (6.398)	30.07	3.74E+06 (6.573)	5.80E+06 (6.763)	-55.18	0.377
12	1.18E+07 (7.071)	1.10E+07 (7.043)	6.36	9.15E+06 (6.961)	1.33E+07 (7.123)	-45.22	0.312
18	1.35E+07 (7.131	1.67E+07 (7.223)	-23.57	1.05E+07 (7.023)	1.17E+07 (7.068)	-11.03	0.228
24	1.72E+07 (7.236)	1.50E+07 (7.177)	12.71	5.58E+06 (6.746)	7.89E+06 (6.897)	-41.48	0.317

*=Least significant difference

**=The values in parenthesis indicate the converted value of number of viable cells (CFU/mL) into log_{10} CFU/mL.

Table S4. Effect of crude *Bl* 1821 putative antibacterial proteins (ABPs) on the OD_{600nm} reading of *Bl* 1821L and *Bl* 1951 after incubation at 30°C for various time intervals. Data presents the mean values of four experiments.

Time intervals (Hours)	*Bl* 1821L	*Bl* 1821L + *Bl* 1821L crude ABPs	% Decrease/increase in OD_{600nm} reading	*Bl* 1951	*Bl* 1951 + *Bl* 1821L crude ABPs	% Decrease/increase in OD_{600nm} reading	*LSD (5%)
1	1.99	1.99	0.00	1.92	1.90	1.14	0.061
3	1.87	1.85	0.67	1.74	1.73	0.43	0.121
6	1.65	1.63	1.21	1.58	1.55	1.51	0.148
12	1.29	1.26	2.32	1.45	1.39	4.57	0.135
18	1.23	1.21	1.63	1.24	1.25	-0.10	0.227
24	1.25	1.18	5.51	1.29	1.18	8.36	0.237
**% Decrease from the start (1 hour) to the end (24 hours) of incubation**	37.2%	40.7%		32.8%	37.9%		

*=Least significant difference

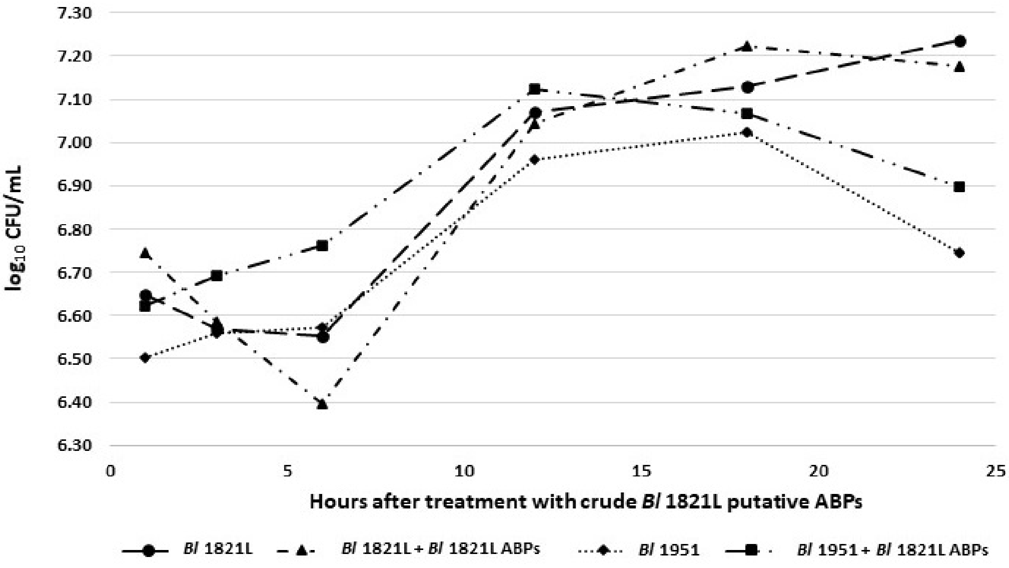

Figure S7. **Number of viable cells (log_{10} CFU/mL) of *Bl* 1821L and *Bl* 1951 with/without treatment of crude *Bl* 1821L putative antibacterial proteins (ABPs) after incubation at 30°C over 24 hours.**

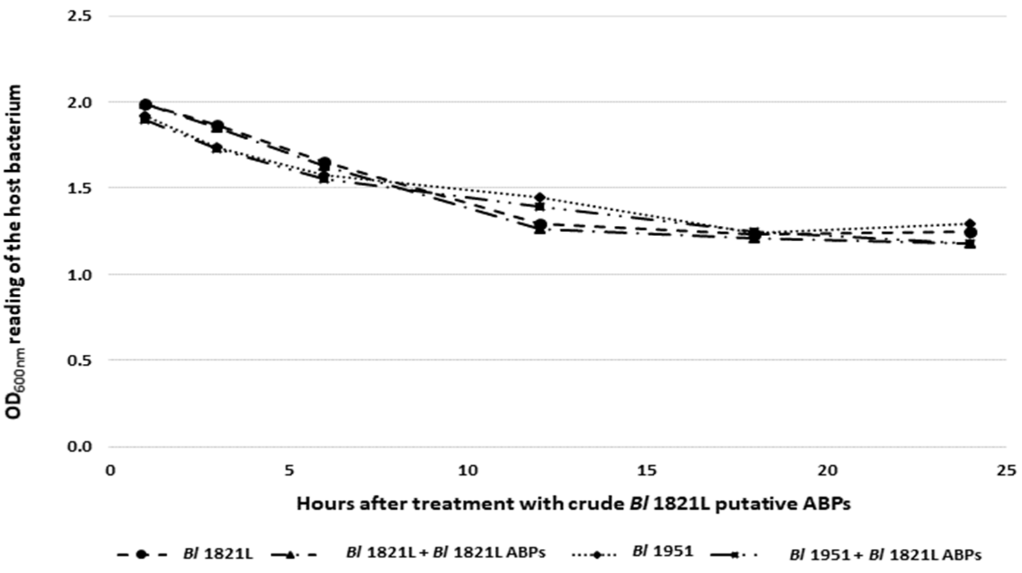

Figure S8. **Effect of crude *Bl* 1821 putative antibacterial proteins (ABPs) on the OD_{600nm} reading of *Bl* 1821L and *Bl* 1951 after incubation at 30°C for various time intervals.**

Table S5. **Effect of purified *Bl* 1821L putative ~48 kD phage tail-like protein (PTLP) on the number of viable cells of *Bl* 1821L and *Bl* 1951 after incubation at 30°C for various time intervals. Data presents the mean values of one experiment. Values of % decrease/increase in the number of viable cells are calculated from CFUs values of corresponding time intervals .**

Time intervals (Hours)	*Bl* 1821L	*Bl* 1821L + *Bl* 1821L PTLP	% Decrease/increase in no. of viable cells	*Bl* 1951	*Bl* 1951 + *Bl* 1821L PTLP	% Decrease/increase in no. of viable cells
1	1.74E+07 (7.241)*	2.81E+07 (7.448)	-61.21	9.05E+06 (6.957)	1.66E+07 (7.220)	-83.43
3	2.41E+07 (7.381)	2.16E+07 (7.334)	10.19	6.20E+06 (6.792)	8.80E+06 (6.944)	-41.94
6	1.13E+07 (7.051)	1.64E+07 (7.215)	-45.78	1.17E+07 (7.066)	1.08E+07 (7.031)	7.73
12	2.34E+07 (7.369)	2.14E+07 (7.330)	8.55	2.58E+07 (7.411)	2.01E+07 (7.302)	22.14
18	1.68E+07 (7.224)	2.78E+07 (7.443)	-65.67	2.37E+07 (7.375)	1.56E+07 (7.193)	34.18
24	2.33E+07 (7.366)	2.87E+07 (7.457)	-23.23	1.87E+07 (7.271)	2.03E+07 (7.307)	-8.85

*= The values in parenthesis indicate the number of viable cells (CFUs/mL) converted into log_{10} CFU/mL.

Table S6. Effect of purified *Bl* 1821L ~48 kD putative phage tail-like protein (PTLP) on the OD_{600nm} reading of *Bl* 1821L and *Bl* 1951 after incubation at 30°C for various time intervals. Data presents the mean values of one experiment.

Time intervals (Hours)	*Bl* 1821L	*Bl* 1821L + *Bl* 1821L PTLP	% Decrease/increase in OD_{600nm} reading	*Bl* 1951	*Bl* 1951 + *Bl* 1821L PTLP	% Decrease/increase in OD_{600nm} reading
1	3.40	3.20	5.88	2.89	2.90	-0.35
3	1.99	1.98	0.50	1.93	1.91	1.04
6	1.70	1.61	5.01	1.76	1.79	-1.70
12	1.26	1.30	-3.59	1.65	1.68	-1.82
18	1.19	1.27	-6.30	1.58	1.59	-0.63
24	1.14	1.21	-6.14	1.43	1.50	-4.90
**% Decrease from the start (1 hour) to the end (24 hours) of incubation**	66.5%	62.2%			50.5%	48.3%

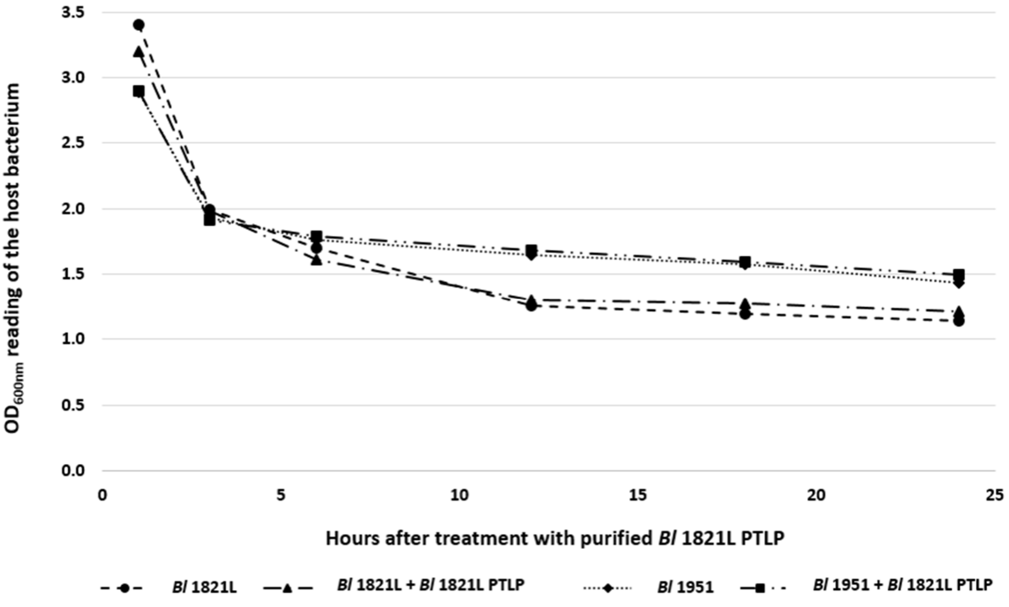

Figure S9. Effect of purified *Bl* 1821 putative phage tail-like protein (~48 kD) on the OD_{600nm} reading of *Bl* 1821L and *Bl* 1951 after incubation at 30°C for various time intervals.

Table S7. Phage-like element PBSX protein XkdK and phage tail-sheath proteins from different gram-positive bacteria.

Accession	Protein name	Organism
A0A0D1WNL8	Phage tail sheath	*Aneurinibacillus migulanus*
A0A410KN98I	Phage tail sheath	*Bacillus aerophilus*
R4JQA6	Structural protein	*Bacillus* phage PBP180
A0A5B0B6Z4	Phage-like element PBSX XkdK	*Bacillus* sp. ANT_WA51
A0A410QZ71	Phage-like element PBSX XkdK	*Bacillus* sp. WR11
A0A6H0H1P2	Phage-like element PBSX XkdK	*Bacillus subtilis* subsp. *subtilis* str. SMY
P54331	Phage-like element PBSX XkdK	*Bacillus subtilis168*
C0Z5G9	Uncharacterised protein	*Brevibacillus brevis* (strain 47/JCM 6285/ NBRC 100599)
A0A3M8B733	Phage tail	*Brevibacillus gelatini*
A0A075R9L5	Phage tail sheath	*Brevibacillus laterosporus* LMG 15441
Q18BN0	Phage-like element PBSX XkdK	*Clostridioides difficile* 630
A0A061P351	Phage-like element PBSX XkdK	*Geomicrobium* sp. JCM 19039

Figure S10. Amino acids alignment of identified *Bl* 1821L and *Bl* 1951 phage like-element PBSX protein XkdK (A0A518VEB0) with the similar proteins of other gram-positive bacteria (See SM Table 7) using the programme CLUSTALO.

```
A0A0D1WNL8  A0A0D1WNL8_ANEMI  1    ----------MAGGTFQTGERKVRPGFYARFISAAQDRIAVAPRGTVILPLTLNWGRAKE 50
A0A3M8B733  A0A3M8B733_9BACL  1    -----------------MTIQRERPGVTVELIAKAKERV-VPKSGVVLVPYQAEWGAPDE 42
A0A5B0B6Z4  A0A5B0B6Z4_9BACI  1    ----------MNGGTFTTGKEKERAGIYFNFKTTAQERVSLSERGTVALPVASSWGEAKT 50
A0A6H0H1P2  A0A6H0H1P2_BACIU  1    ----------MNGGTFTTGKEKERAGIYFNFKTTAQERVSLSERGTVALPVASSWGEAKT 50
A0A061P351  A0A061P351_9BACL  1    ----------MNGGTFAPGVEKERAGIYFRFTSAANDRLSVGERGTVALPLELSWGAPKT 50
A0A075R9L5  A0A075R9L5_BRELA  1    MKNKYDNTYLHFEEVNSMTIQRERPGVNVELKAKAQERV-LPKSGVVLVPYLAEWGAPDQ 59
A0A410KN98  A0A410KN98_9BACI  1    ----------MNGGTFTPGTEKKRPGIYFNFKTTAEQRITLGERGTVALPLVMSWGEPKT 50
A0A410QZ71  A0A410QZ71_9BACI  1    ----------MNGGTFTTGKEKERAGIYFNFKTTAQERVSLSERGTVALPVASSWGEAKT 50
A0A518VEB0  A0A518VEB0_BRELA  1    -----------------MTIQRERPGVNVELKAKAQERV-LPKSGVVLVPYLAEWGAPDQ 42
C0Z5G9      C0Z5G9_BREBN      1    -----------------MTIQRERPGVTVELIAKAKERV-VPKSGVVLVPYQAEWGAPDE 42
Q18BN0      Q18BN0_CLOD6      1    ------------------------------------------------------------ 0
R4JQA6      R4JQA6_9CAUD      1    ----------MNGGTFTPGTEKKRPGIYFNFKTTAEQRITLGERGTVALPLVMSWGEPKT 50
P54331      XKDK_BACSU        1    ----------MNGGTFTTGKEKERAGIYFNFKTTAQERVSLSERGTVALPVASSWGEAKT 50

A0A0D1WNL8  A0A0D1WNL8_ANEMI  51   FTTIEVEKDTMDKLGYDYNDPEMLLIREARKLAKKVKVYKLNKGEKAKGTFGTT------ 104
A0A3M8B733  A0A3M8B733_9BACL  43   LVKLGSYEERMTETF-----GKVDTVELAAEGGATILAYRMTNGNASKAVYEQA------ 91
A0A5B0B6Z4  A0A5B0B6Z4_9BACI  51   FVSISSVEDLNKKVGLSIDDPSLLLLREAKKNAKTVLMYRLTEGVRASADIA-------- 102
A0A6H0H1P2  A0A6H0H1P2_BACIU  51   FVSISSVEDLNKKVGLSIDDPSLLLLREAKKNAKTVLMYRLTEGVRASADIA-------- 102
A0A061P351  A0A061P351_9BACL  51   FVEINGPDDVLKKYGLSVNDSSVLLLKEAMKRSQTVLAYRVNEGSKAETVIGESSGGGGS 110
A0A075R9L5  A0A075R9L5_BRELA  60   VITMKGYEERVAETF-----GQIDILELAAEGGATVVGYRMTNGKSVAASYSQE------ 108
A0A410KN98  A0A410KN98_9BACI  51   FISVSDMEDLNKKVGLNIDDKSLLLFREAKKKAQTVLLYRLNEGEPAKAEIA-------- 102
A0A410QZ71  A0A410QZ71_9BACI  51   FVSISSVEDLNKKVGLSIDDPSLLLLREAKKNAKTVLMYRLTEGVRASADIA-------- 102
A0A518VEB0  A0A518VEB0_BRELA  43   VITMKGYEERVAETF-----GHIGILELAAEGGATVLGYRMTSGNGVAASYTQS------ 91
C0Z5G9      C0Z5G9_BREBN      43   LVKLGSFEERIAQTF-----GKVDTVELAAEGGATILAYRMTNGTATKAAYEQA------ 91
Q18BN0      Q18BN0_CLOD6      1    ---------------MAIGLPSINI----------------------------------- 10
R4JQA6      R4JQA6_9CAUD      51   FISVSDMEDLNKKVGLNIDDKSLLLFREAKKKAQTVLLYRLNEGEPAKAEIA-------- 102
P54331      XKDK_BACSU        51   FVSISSVEDLNKKVGLSIDDPSLLLLREAKKNAKTVLMYRLTEGVRASADIA-------- 102
                                                         :

A0A0D1WNL8  A0A0D1WNL8_ANEMI  105  -------------SICTVEAINDGTRGNDITIVSQVNVLDTTKKDVITYVKGRQVDKQTQ 151
A0A3M8B733  A0A3M8B733_9BACL  92   -------------DAIRIEALYPGLLGNDLRIVISASTSEPGKKELQVKG-PLQTEKFSF 137
A0A5B0B6Z4  A0A5B0B6Z4_9BACI  103  -------------EGVKATAVYGGTKGNDIIIRINQNVLDANSFDVTTYMDESEVDKQTV 149
A0A6H0H1P2  A0A6H0H1P2_BACIU  103  -------------EGVKATAVYGGTKGNDIIIRINQNVLDANSFDVTTYMDESEVDKQTV 149
A0A061P351  A0A061P351_9BACL  111  DGDDEEEGASGQSNGLRAVAKFGGMKGNDIQIRVSENVLDSELFDVTTYLNNVAVNRQSV 170
A0A075R9L5  A0A075R9L5_BRELA  109  -------------GSIAIQARYPGLVGNELQISIKDSTAELGKKELQVKG-PIKTEKFSF 154
A0A410KN98  A0A410KN98_9BACI  103  -------------ENFVVTANYGGQKGNEITIQVAENVLDSTKRDVITYLGTDIVDKQVV 149
A0A410QZ71  A0A410QZ71_9BACI  103  -------------EGVKATAVYGGTKGNDIIIRINQNVLDANSFDVTTYMDESEVDKQTV 149
A0A518VEB0  A0A518VEB0_BRELA  92   -------------DSFTLEARYPGLVGNDLQISIKESTVELGKKELQVKG-PIKTEKFSF 137
C0Z5G9      C0Z5G9_BREBN      92   -------------DAIRVEALYPGLVGNELKVTITVSTSEPGKKELQVTG-PLQTEKFSF 137
Q18BN0      Q18BN0_CLOD6      11   --------------SFKELA-------TTVKERSARGIIAMVLKDAKALGLNEIHEKEDI 49
R4JQA6      R4JQA6_9CAUD      103  -------------ENFVVTANYGGQKGNEITIQVAENVLDSTKRDVITYLGTDIVDKQVV 149
P54331      XKDK_BACSU        103  -------------EGVKATAVYGGTKGNDIIIRINQNVLDANSFDVTTYMDESEVDKQTV 149
                                                      *       . :      .       :  .       ::
```

```
A0A0D1WNL8  A0A0D1WNL8_ANEMI 152   AEIESL----VPNEWVTFSGTGAIEA------------------TAGTTLTGGTNGTVIN   189
A0A3M8B733  A0A3M8B733_9BACL 138   ANADELVEKIGQSVYVRVQKLGDAA----------------IATAAETNLTGGTSGTVAL   181
A0A5B0B6Z4  A0A5B0B6Z4_9BACI 150   KKAEEL----TANGYVTFTGTGDLSSTIPLTGSEGDTAAETLNASAGIRLSGGTDKAPVN   205
A0A6H0H1P2  A0A6H0H1P2_BACIU 150   KKAEEL----TANGYVTFTGTGDLSSTIPLTGSEGDTAAETLNASAGIRLSGGTDKAPVN   205
A0A061P351  A0A061P351_9BACL 171   RRSSEL----QDNDWVSFEGSGRLQE------------------TAGTNLSGGTNSEART   208
A0A075R9L5  A0A075R9L5_BRELA 155   ANMDELVTKAEQSIYIKVKKLGDKA----------------VEETAMTALSGGTSGIATL   198
A0A410KN98  A0A410KN98_9BACI 150   KDVKDL----VKNKYVQFSGEGEAVI------------------TAGAALSGGKNGVASV   187
A0A410QZ71  A0A410QZ71_9BACI 150   KKAEEL----TANGYVTFTGTGDLSSTIPLTGSEGDTAAETLNASAGIRLSGGTDKAPVN   205
A0A518VEB0  A0A518VEB0_BRELA 138   ANVDELVTKAEQSIYIKVKKLGDKV----------------AEETMMISLEGGTSGITSL   181
C0Z5G9      C0Z5G9_BREBN     138   ADANELAAKTSQSNYVRVKKLGETA----------------VTIVPETALTGAKSGTVAL   181
Q18BN0      Q18BN0_CLOD6      50   PVDLSA----ENKEYINLALMGNVNT-------------------PNKLLVYVIEGEADI    86
R4JQA6      R4JQA6_9CAUD     150   KDVKDL----VKNKYVQFSGEGEAVI------------------TAGAALSGGKNGVASV   187
P54331      XKDK_BACSU       150   KKAEEL----TANGYVTFTGTGDLSSTIPLTGSEGDTAAETLNASAGIRLSGGTDKAPVN   205
                                       .       . :: .   *                           *    .

A0A0D1WNL8  A0A0D1WNL8_ANEMI 190   --ADYTDFMTACETEFFDTIGFLSD-DVTLKTTFVGFIRRLREDEGRKVKGVVAGYEADY   246
A0A3M8B733  A0A3M8B733_9BACL 182   AAADATKLFMAVSGADFDAMYLPFDD-PAVQAAAKQFMSDRRTLGKKLSTLVIGGKAVED   240
A0A5B0B6Z4  A0A5B0B6Z4_9BACI 206   --SDYTDFLAAAETESFDVIALPVAEGDQLKATFAAFIKRLRDGQGQKVQGVTANYAGDY   263
A0A6H0H1P2  A0A6H0H1P2_BACIU 206   --SDYTDFLAAAETESFDVIALPVAEGDQLKATFAAFIKRLRDGQGQKVQGVTANYAGDY   263
A0A061P351  A0A061P351_9BACL 209   --WDYTDFLSAAEHVSYDTIGLPVDDEDQLKTTFVSFVKRMRDETGIKITGVLPNFDGDH   266
A0A075R9L5  A0A075R9L5_BRELA 199   SATDFTTLFNSISGVDFDAMYLPSAD-AGIQAAAKQFMVDRELFSKKRSTLVIGGLPEKD   257
A0A410KN98  A0A410KN98_9BACI 188   --ADYTAFLEAAETEYFDVIALPVDNSEQLKATFASFIERLRDKQGRKVQGVVANYAADQ   245
A0A410QZ71  A0A410QZ71_9BACI 206   --SDYTDFLAAAETESFDVIALPVAEGDQLKATFAAFIKRLRDGQGQKVQGVTANYAGDY   263
A0A518VEB0  A0A518VEB0_BRELA 182   TANDFTTLFNSISGTDFDAMYLPSAD-VAIQAAAKQFMSDRELFSKKRSTLVIGGLKDKD   240
C0Z5G9      C0Z5G9_BREBN     182   ASADSTKLFMAVSGADFDAMYLPFDD-SAVQAAAKQFMSDRRTQNKKLSTLVIGGKAADD   240
Q18BN0      Q18BN0_CLOD6      87   --QTALDFL---ETKEFNYLCMPKA-VEADKTAIKNWIIKLRDIDKVKVKAVLGKVVGNH   140
R4JQA6      R4JQA6_9CAUD     188   --ADYTAFLEAAETEYFDVIALPVDNSEQLKATFASFIERLRDKQGRKVQGVVANYAADQ   245
P54331      XKDK_BACSU       206   --SDYTDFLAAAETESFDVIALPVAEGDQLKATFAAFIKRLRDGQGQKVQGVTANYAGDY   263
                                          ::   .   :: : :        :::   ::   .         *      .

A0A0D1WNL8  A0A0D1WNL8_ANEMI 247   ---------------EGIINVKNSVKLLDGTELTKERAVAWVAGADAGASITKSNTYQRY   291
A0A3M8B733  A0A3M8B733_9BACL 241   ENMEKHVERSVAQNARFVVNCAIAGQHNNGNTYGSLEWAAWVAGMIAATPAHESLTAVVV   300
A0A5B0B6Z4  A0A5B0B6Z4_9BACI 264   ---------------EGIINVTEGVLLEDGTEVTPDKATAWVAGASAGATFNQSLTFVEY   308
A0A6H0H1P2  A0A6H0H1P2_BACIU 264   ---------------EGIINVTEGVLLEDGTEVTPDKATAWVAGASAGATFNQSLTFVEY   308
A0A061P351  A0A061P351_9BACL 267   ---------------EGIINVTSSVELAER-KLNVAETVAWVAGASAGATINQSLTFVDY   310
A0A075R9L5  A0A075R9L5_BRELA 258   SNMNEHVDRSAANNSRRVVNCAIAGQHVNGKTYGSLEWAAWLAGMIAATPAHISLSAQLV   317
A0A410KN98  A0A410KN98_9BACI 246   ---------------EGIINVTSGVVLEDGTELTPAQTTAWVAGASAGANFNQSLTFVEY   290
A0A410QZ71  A0A410QZ71_9BACI 264   ---------------EGIINVTEGVLLEDGTEVTPDKATAWVAGASAGATFNQSLTFVEY   308
A0A518VEB0  A0A518VEB0_BRELA 241   NNMNEHVQRSVANNSRRVVNCAIAGQHVNGKTYGSLEWAAWLAGMIAATPAHISLSAQLV   300
C0Z5G9      C0Z5G9_BREBN     241   ENMAKHIERSVAQNARFVVNSAIAGQHNNGKVYGSLEWAAWVAGMIAATPAHESLTAVVV   300
Q18BN0      Q18BN0_CLOD6     141   ---------------EGIINFTTEDVLVGEKKYSVDEFTSRVAGLIAGTPLSQSVTYTKL   185
R4JQA6      R4JQA6_9CAUD     246   ---------------EGIINVTSGVVLEDGTELTPAQTTAWVAGASAGANFNQSLTFVEY   290
P54331      XKDK_BACSU       264   ---------------EGIINVTEGVLLEDGTEVTPDKATAWVAGASAGATFNQSLTFVEY   308
                                                  . ::*                . .: :**  *.:    * :
```

```
A0A0D1WNL8  A0A0D1WNL8_ANEMI 292   DGAVDAIPRYTNSEIIEAINKGEFVFVNDG--EQVKVEYDINSLVTTGQDKNSRFKKNRV   349
A0A3M8B733  A0A3M8B733_9BACL 301   PL-KKAQKDWGHSDILRALGSGTLVATRDG--DVYIIESAVNTLSVLGNHEREDYGKIRV   357
A0A5B0B6Z4  A0A5B0B6Z4_9BACI 309   EGAVDVLHRLDHDTIVERLGKGEFLFTFDARDKSVSVEKDINSLVTFTAEKNKKFAKNKI   368
A0A6H0H1P2  A0A6H0H1P2_BACIU 309   EGAVDVLHRLDHDTIVERLGKGEFLFTFDARDKSVSVEKDINSLVTFTAEKNKKFAKNKI   368
A0A061P351  A0A061P351_9BACL 311   EGAIGVNEDLDDDEIKERLRNGEFLFHYDPRDRTVSVEKDINSFTSFTAEKNYRFSKNLI   370
A0A075R9L5  A0A075R9L5_BRELA 318   PM-KKAAKDWGHTEIQNALNSGSLIAVRDG--DVYLIESAVNTLTTLKAAEREDFGKIRV   374
A0A410KN98  A0A410KN98_9BACI 291   EGAVDTLERLDNDQVEYRLSQGEFLFTFDARDRTVSVEKDINSLTSFTVEKNQQMAKNKI   350
A0A410QZ71  A0A410QZ71_9BACI 309   EGAVDVLHRLDHDTIVERLGKGEFLFTFDARDKSVSVEKDINSLVTFTAEKNKKFAKNKI   368
A0A518VEB0  A0A518VEB0_BRELA 301   PL-KKAEKDWGHTDIQAALNSGTLIAVRDG--DVYLIESAVNTLTTLKAAEREDFGKIRV   357
C0Z5G9      C0Z5G9_BREBN     301   PL-KKALKDWGHTDILSALGSGTLIATRDG--DVYIIESAVNTLAVLGTHEREDYGKIRV   357
Q18BN0      Q18BN0_CLOD6     186   SDVVDI-PKMTKVDAESKVNKGELILIKEA--GAIRIARGVNSLTELTAEKGEMFQKIKI   242
R4JQA6      R4JQA6_9CAUD     291   EGAVDTLERLDNDQVEYRLSQGEFLFTFDARDRTVSVEKDINSLTSFTVEKNQQMAKNKI   350
P54331      XKDK_BACSU       309   EGAVDVLHRLDHDTIVERLGKGEFLFTFDARDKSVSVEKDINSLVTFTAEKNKKFAKNKI   368
                                              .     : .*::   :       :   :*::        :     *  :

A0A0D1WNL8  A0A0D1WNL8_ANEMI 350   MRTLDAIHNDIKREIERNYIG------KMNNNADGQAILKASVIGYLETLQNANAIQNLD   403
A0A3M8B733  A0A3M8B733_9BACL 358   SMTLDQIVNDINAVGK-KYKG------KLGNNDLGGAVFVSAVNAYMTVREQQGAIEPGW   410
A0A5B0B6Z4  A0A5B0B6Z4_9BACI 369   VRVLDAVNNDLTRELKALIKSRKGSGSDIPASEDGLQYVKTMITQYMTTLQDAGGITGFD   428
A0A6H0H1P2  A0A6H0H1P2_BACIU 369   VRVLDAVNNDLTRELKALIKSRKGSGSDIPASEDGLQYVKTMITQYMTTLQDAGGITGFD   428
A0A061P351  A0A061P351_9BACL 371   VRVLDAINNDITRELKALIKLRKDRGQDIPGSNDGKQIIRSLITVYLNALQEGGAIKNFD   430
A0A075R9L5  A0A075R9L5_BRELA 375   SMTLDQIVNDITSVGK-KYKG------KLDNNDIGGATFVGAVKTYLEVREAQGAIDKGW   427
A0A410KN98  A0A410KN98_9BACI 351   IRVLDAINNDLTFELKNLIKLRKANGNDIPASDDGVQLVKTLITQYLTQLQDGSGITGFN   410
A0A410QZ71  A0A410QZ71_9BACI 369   VRVLDAVNNDLTRELKALIKSRKGSGSDIPASEDGLQYVKTMITQYMTTLQDAGGITGFD   428
A0A518VEB0  A0A518VEB0_BRELA 358   SMTLDQIVNDITSVGK-KYKG------KLDNNDIGGATFVGAVKTYLEVREAQGAIDQGW   410
C0Z5G9      C0Z5G9_BREBN     358   SMTLDQIVNDISQVGK-KYKG------KLGNNDLGGAVFVSAVNAYMTVREQQGAIDTGW   410
Q18BN0      Q18BN0_CLOD6     243   VDTLDIIHSDIRKVIIDDYIG------KVTNSYDNKCLLIVAIKSYLEELEKSALIES-D   295
R4JQA6      R4JQA6_9CAUD     351   IRVLDAINNDLTFELKNLIKLRKANGNDIPASDDGVQLVKTLITQYLTQLQDGSGITGFN   410
P54331      XKDK_BACSU       369   VRVLDAVNNDLTRELKALIKSRKGSGSDIPASEDGLQYVKTMITQYMTTLQDAGGITGFD   428
                                    .** :  .*:                .:   .   .    .    :  *:    :    *

A0A0D1WNL8  A0A0D1WNL8_ANEMI 404   KDADFVIDPVLSV---------------------DDEVHATIYVQPVDSMEKFYFTILVR   442
A0A3M8B733  A0A3M8B733_9BACL 411   TFTDK-------------------------KNGNGDRRGFQLSAKPLDAIEYFDIDWEVL   445
A0A5B0B6Z4  A0A5B0B6Z4_9BACI 429   SDEDITISMNE-D---------------------RDGFLIDLAVQPVDAAEKFYFNVEVN   466
A0A6H0H1P2  A0A6H0H1P2_BACIU 429   SDEDITISMNE-D---------------------RDGFLIDLAVQPVDAAEKFYFNVEVN   466
A0A061P351  A0A061P351_9BACL 431   PSDDIDIVLTD-Q---------------------EDGFIINLGAQPVDAAEKFYFDTEVR   468
A0A075R9L5  A0A075R9L5_BRELA 428   IFEDK-------------------------KNGIGDKRGFRLAAKPLDAIELFDIEWEVL   462
A0A410KN98  A0A410KN98_9BACI 411   SETDIVIGLNE-D---------------------RDGFIIDLAVQPVDAAEKFYFNVEVK   448
A0A410QZ71  A0A410QZ71_9BACI 429   SDEDITISMNE-D---------------------RDGFLIDLAVQPVDAAEKFYFNVEVN   466
A0A518VEB0  A0A518VEB0_BRELA 411   IFEDK-------------------------KNGIGDKRGFRLAAKPLDAIEIFDIEWEVL   445
C0Z5G9      C0Z5G9_BREBN     411   TFTDK-------------------------KNGIGDRRGFLLSAKPLDAIEYFDIDWEVL   445
Q18BN0      Q18BN0_CLOD6     296   STVEIDFEAQKSYLKSKGVDLSYMTLQEIKEANTGSKVFLKAKIKVLDAMEDIDLSIEI-   354
R4JQA6      R4JQA6_9CAUD     411   SETDIVIGLNE-D---------------------RDGFIIDLAVQPVDAAEKFYFNVEVK   448
P54331      XKDK_BACSU       429   SDEDITISMNE-D---------------------RDGFLIDLAVQPVDAAEKFYFNVEVN   466
                                     :                                .         : :*:  * : :   :
```

*Highlighted dark grey color amino acids denote similarity among the aligned phage-like element PBSX protein XkdK.
(Refer to SM Tables S8 & S9)

Table S8. Distance matrices of identified *Bl* 1821L and *Bl* 1951 phage-like element PBSX protein XkdK (A0A518VEB0) with the similar proteins of other gram-positive bacteria.

Uniprot Accession # (www.uniprot.org)	Uniprot Accession # (https://www.uniprot.org)												
	A0A0D1WNL8	A0A3M8B733	A0A5B0B6Z4	A0A6H0H1P2	A0A061P351	A0A075R9L5	A0A410KN98	A0A410QZ71	A0A518VEB0	C0Z5G9	Q18BN0	R4JQA6	P54331
**A0A0D1WNL8**		1.34	0.8	0.8	0.84	1.4	0.8	0.8	1.33	1.46	1.51	0.8	0.8
**A0A3M8B733**	1.34		1.44	1.44	1.43	0.38	1.4	1.44	0.36	0.14	1.64	1.4	1.44
**A0A5B0B6Z4**	0.8	1.44		0	0.56	1.52	0.37	0	1.45	1.45	1.49	0.37	0
**A0A6H0H1P2**	0.8	1.44	0		0.56	1.52	0.37	0	1.45	1.45	1.49	0.37	0
**A0A061P351**	0.84	1.43	0.56	0.56		1.42	0.57	0.56	1.45	1.38	1.54	0.57	0.56
**A0A075R9L5**	1.4	0.38	1.52	1.52	1.42		1.44	1.55	0.1	0.39	1.49	1.44	1.55
**A0A410KN98**	0.8	1.4	0.37	0.37	0.57	1.44		0.37	1.4	1.37	1.66	0	0.37
**A0A410QZ71**	0.8	1.44	0	0	0.56	1.55	0.37		1.45	1.45	1.49	0.37	0
**A0A518VEB0**	1.33	0.36	1.45	1.45	1.45	0.1	1.4	1.45		0.39	1.52	1.4	1.45
**C0Z5G9**	1.46	0.14	1.45	1.45	1.38	0.39	1.37	1.45	0.39		1.66	1.37	1.45
**Q18BN0**	1.51	1.64	1.49	1.49	1.54	1.49	1.66	1.49	1.52	1.66		1.66	1.49
**R4JQA6**	0.8	1.4	0.37	0.37	0.57	1.44	0	0.37	1.4	1.37	1.66		0.37
**P54331**	0.8	1.44	0	0	0.56	1.55	0.37	0	1.45	1.45	1.49	0.37	

Table S9. Amino acids alignment % of identified *Bl* 1821L and *Bl* 1951 phage-like element PBSX protein XkdK (A0A518VEB0) with the similar proteins of other gram-positive bacteria.

Uniprot Accession # (www.uniprot.org)	Uniprot Accession # (https://www.uniprot.org)												
	**A0A0D1WNL8**	**A0A3M8B733**	**A0A5B0B6Z4**	**A0A6H0H1P2**	**A0A061P351**	**A0A075R9L5**	**A0A410KN98**	**A0A410QZ71**	**A0A518VEB0**	**C0Z5G9**	**Q18BN0**	**R4JQA6**	**P54331**
**A0A0D1WNL8**		23.9	41.5	41.5	39.8	22.0	42.6	41.5	22.7	23.2	22.0	42.6	41.5
**A0A3M8B733**	23.9		21.2	21.2	20.7	68.2	20.6	21.2	69.0	86.0	18.4	20.6	21.2
**A0A5B0B6Z4**	41.5	21.2		100.0	51.8	20.3	64.8	100.0	21.2	20.5	22.4	64.8	100.0
**A0A6H0H1P2**	41.5	21.2	100.0		51.8	20.3	64.8	100.0	21.2	20.5	22.4	64.8	100.0
**A0A061P351**	39.8	20.7	51.8	51.8		19.0	53.4	51.8	18.8	19.4	20.5	53.4	51.8
**A0A075R9L5**	22.0	68.2	20.3	20.3	19.0		19.8	20.3	89.1	67.4	19.3	19.8	20.3
**A0A410KN98**	42.6	20.6	64.8	64.8	53.4	19.8		64.8	20.3	22.0	20.1	100.0	64.8
**A0A410QZ71**	41.5	21.2	100.0	100.0	51.8	20.3	64.8		21.2	20.5	22.4	64.8	100.0
**A0A518VEB0**	22.7	69.0	21.2	21.2	18.8	89.1	20.3	21.2		68.1	19.9	20.3	21.2
**C0Z5G9**	23.2	86.0	20.5	20.5	19.4	67.4	22.0	20.5	68.1		20.2	22.0	20.5
**Q18BN0**	22.0	18.4	22.4	22.4	20.5	19.3	20.1	22.4	19.9	20.2		20.1	22.4
**R4JQA6**	42.6	20.6	64.8	64.8	53.4	19.8	100.0	64.8	20.3	22.0	20.1		64.8
**P54331**	41.5	21.2	100.0	100.0	51.8	20.3	64.8	100.0	21.2	20.5	22.4	64.8	

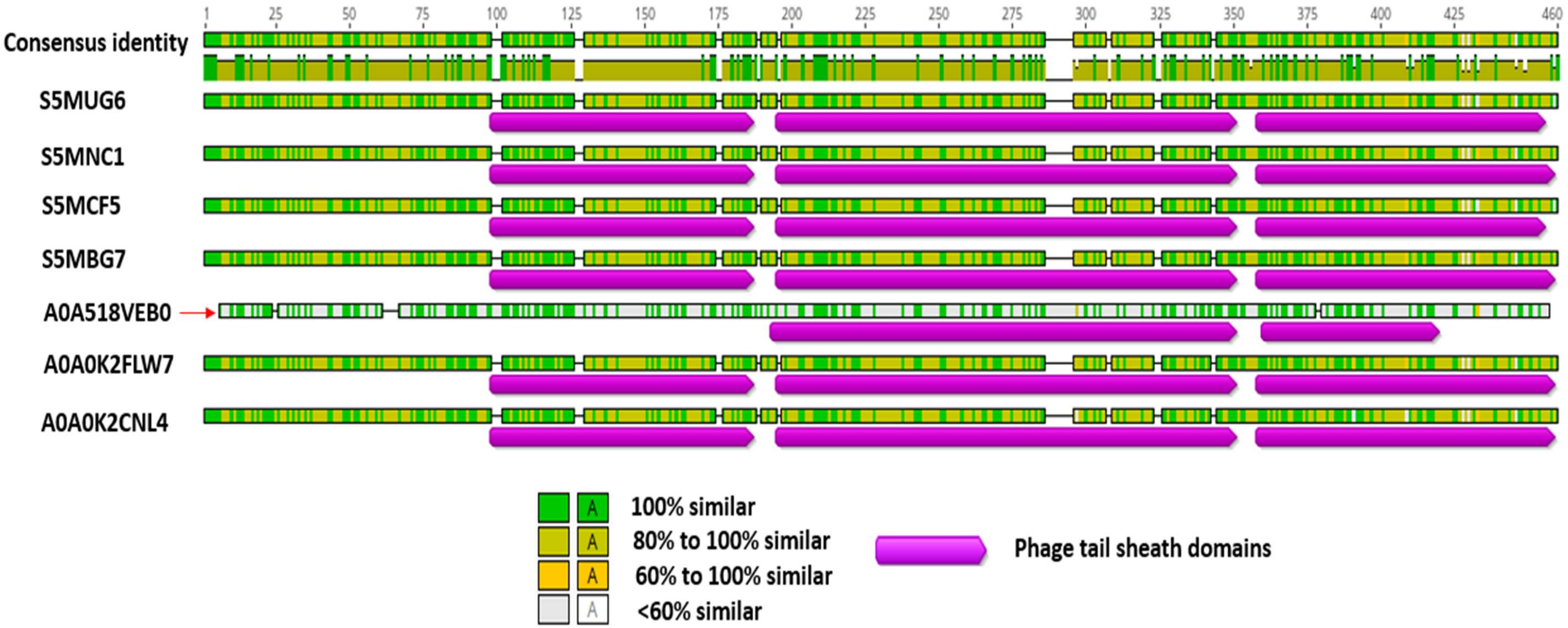

Figure S11.Amino acids alignment and percentage identity of identified putative phage tail-sheath protein (A0A518VEB0, shown with red arrow) of *Bl* 1821L and *Bl* 1951 with similar proteins of different *Bl* phages including Abouo (S5MUG6), Jimmer1 (S5MNC1), Davies (S5MCF5), Jimmer2, (S5MBG7), Powder (A0A0K2FLW7), and Osiris (A0A0K2CNL4) using Geneious basic.

(Refer to SM Figure S12 & Tables S10 and S11)

Figure S12. Amino acids alignment of identified *Bl* 1821L and *Bl* 1951 putative phage tail-sheath protein (A0A518VEB0) with the phage tail-sheath protein of different *Bl* phages using the programme CLUSTALO.

```
A0A518VEB0  A0A518VEB0_BRELA     1  -----MTIQRERPGVNVELKAKA-QERVLPKSGVVLVPYLAEWGAPDQVITMKGYEERVA  54
S5MUG6      S5MUG6_9CAUD         1  MMQNWIAQNKVRPGTYVNVVSEAKPQGAISERGTVTMALSLSWGPSGEVIEIQAGENTLD  60
S5MNC1      S5MNC1_9CAUD         1  MMQNWIAQNKVRPGTYVNVVSEAKPQGAISERGTVTMALSLSWGPSGEVIEIQAGENTLD  60
S5MCF5      S5MCF5_9CAUD         1  MMQNWIAQNKVRPGTYVNVVSEAKPQGAISERGTVTMALSLSWGPSGEVIEIQAGENTLD  60
S5MBG7      S5MBG7_9CAUD         1  MMQNWIAQNKVRPGTYVNVVSEAKPQGAISERGTVTMALSLSWGPSGEVIEIQAGENTLD  60
A0A0K2FLW7  A0A0K2FLW7_9CAUD     1  MMQNWIAQNKVRPGTYVNVVSEAKPQGAISERGTVTMALSLSWGPSGEVIEIQAGENTLD  60
A0A0K2CNL4  A0A0K2CNL4_9CAUD     1  MMQNWIAQNKVRPGTYVNVVSEAKPQGAISERGTVTMALSLSWGPSGEVIEIQAGENTLD  60
                                         :: :: ***. *:: ::*  : .: : *.* :    .**  .:** ::. *: : 

A0A518VEB0  A0A518VEB0_BRELA    55  ETF-----GHIGILELAAEGGATVLGYRMTSGNGVAASYTQSDSFTLEARYPGLVGNDLQ  109
S5MUG6      S5MUG6_9CAUD        61  KLGYDISEPQILLVQEALKRAKTLLLYRLNTGSKAQ---ASSDNLTVTALYGGVRGNDIT  117
S5MNC1      S5MNC1_9CAUD        61  KLGYDISEPQILLVQEALKRAKTLLLYRLNTGSKAQ---ASSDNLTVTALYGGVRGNDIT  117
S5MCF5      S5MCF5_9CAUD        61  KLGYDISEPQILLVQEALKRAKTLLLYRLNTGSKAQ---ASSDNLTVTALYGGVRGNDIT  117
S5MBG7      S5MBG7_9CAUD        61  KLGYDISEPQILLVQEALKRAKTLLLYRLNTGSKAQ---ASSDNLTVTALYGGVRGNDIT  117
A0A0K2FLW7  A0A0K2FLW7_9CAUD    61  KLGYDISEPQILLVQEALKRAKTLLLYRLNTGSKAQ---ASSDNLTVTALYGGVRGNDIT  117
A0A0K2CNL4  A0A0K2CNL4_9CAUD    61  KLGYDISEPQILLVQEALKRAKTLLLYRLNTGSKAQ---ASSDNLTVTALYGGVRGNDIT  117
                                    :        :* ::: * : . *:* **:.:*. .    :.**.:*: * * *: ***:

A0A518VEB0  A0A518VEB0_BRELA   110  ISIKESTVELGKK--ELQVKGPIKTEKFSFANVDELVTKAEQSIYIKVKKLGDKVAEETM  167
S5MUG6      S5MUG6_9CAUD       118  VVVEQNIDDETTFIVKTLVAGSIVDNQLA-----KKIEDLKANKFVTFSGTGALV--ASA  170
S5MNC1      S5MNC1_9CAUD       118  VVVEQNIDDETTFIVKTLVAGSIVDNQLA-----KKIEDLKANKFVTFSGTGALV--ASA  170
S5MCF5      S5MCF5_9CAUD       118  VVVEQNIDDETTFIVKTLVAGSIVDNQLA-----KKIEDLKANKFVTFSGTGALV--ASA  170
S5MBG7      S5MBG7_9CAUD       118  VVVEQNIDDETTFIVKTLVAGSIVDNQLA-----KKIEDLKANKFVTFSGTGALV--ASA  170
A0A0K2FLW7  A0A0K2FLW7_9CAUD   118  VVVEQNIDDETTFIVKTLVAGSIVDNQLA-----KKIEDLKANKFVTFSGTGALV--ASA  170
A0A0K2CNL4  A0A0K2CNL4_9CAUD   118  VVVEQNIDDETTFIVKTLVAGSIVDNQLA-----KKIEDLKANKFVTFSGTGALV--ASA  170
                                    : :::.  :  .   :  * * *  ::::     : : . : . ::...  *  *   :

A0A518VEB0  A0A518VEB0_BRELA   168  MISLEGGTSGITSLTANDFTTLFNSISGTDFDAMYLPSADVAIQAAAKQFMSDRELFSKK  227
S5MUG6      S5MUG6_9CAUD       171  GIPLTGGTDGTEIG--VNHTAYREAIELHDFDAMAVPYDDPTIKSVYVAFAKRLANQQNR  228
S5MNC1      S5MNC1_9CAUD       171  GIPLTGGTDGTEIG--VNHTAYREAIELHDFDAMAVPYDDPTIKSVYVAFAKRLANQQNR  228
S5MCF5      S5MCF5_9CAUD       171  GIPLTGGTDGTEIG--VNHTAYREAIELHDFDAMAVPYDDPTIKSVYVAFAKRLANQQNR  228
S5MBG7      S5MBG7_9CAUD       171  GIPLTGGTDGTEIG--VNHTAYREAIELHDFDAMAVPYDDPTIKSVYVAFAKRLANQQNR  228
A0A0K2FLW7  A0A0K2FLW7_9CAUD   171  GIPLTGGTDGTEIG--VNHTAYREAIELHDFDAMAVPYDDPTIKSVYVAFAKRLANQQNR  228
A0A0K2CNL4  A0A0K2CNL4_9CAUD   171  GIPLTGGTDGTEIG--VNHTAYREAIELHDFDAMAVPYDDPTIKSVYVAFAKRLANQQNR  228
                                     * * ***.*       :.*:  ::*.  ***** :*  * :*::.   * .     .::
```

```
A0A518VEB0  A0A518VEB0_BRELA    228    RSTLVIGGLKDKDNNMNEHVQRSVANNSRRVVNCAIAGQHVNGKTYGSLEWAAWLAGMIA        287
S5MUG6      S5MUG6_9CAUD        229    FIQIVVPNYAQADDPT--------------VISVSNGVILSNSTVIDAVKATAWVAGATA        274
S5MNC1      S5MNC1_9CAUD        229    FIQIVVPNYAQADDPT--------------VISVSNGVILSNSTVIDAVKATAWVAGATA        274
S5MCF5      S5MCF5_9CAUD        229    FIQIVVPNYAQADDPT--------------VISVSNGVILSNSTVIDAVKATAWVAGATA        274
S5MBG7      S5MBG7_9CAUD        229    FIQIVVPNYAQADDPT--------------VISVSNGVILSNSTVIDAVKATAWVAGATA        274
A0A0K2FLW7  A0A0K2FLW7_9CAUD    229    FIQIVVPNYAQADDPT--------------VISVSNGVILSNSTVIDAVKATAWVAGATA        274
A0A0K2CNL4  A0A0K2CNL4_9CAUD    229    FIQIVVPNYAQADDPT--------------VISVSNGVILSNSTVIDAVKATAWVAGATA        274
                                          :*: .  : *:                *:. : .    *... .::: :**:**  *

A0A518VEB0  A0A518VEB0_BRELA    288    ATPAHISLSAQLVPL-KKAEKDWGHTDIQAALNSGTLIAVRDGDVYLIESAVNTLTTLKA        346
S5MUG6      S5MUG6_9CAUD        275    GANVNQSLTHTAYDDAVAVHGRLNDSQITKALLNGEFLFELHNGKVVVEQDINTFTSFSP        334
S5MNC1      S5MNC1_9CAUD        275    GANVNQSLTHTAYDDAVAVHGRLNDSQITKALLNGEFLFELHNGKVVVEQDINTFTSFSP        334
S5MCF5      S5MCF5_9CAUD        275    GANVNQSLTHTAYDDAVAVHGRLNDSQITKALLNGEFLFELHNGKVVVEQDINTFTSFSP        334
S5MBG7      S5MBG7_9CAUD        275    GANVNQSLTHTAYDDAVAVHGRLNDSQITKALLNGEFLFELHNGKVVVEQDINTFTSFSP        334
A0A0K2FLW7  A0A0K2FLW7_9CAUD    275    GANVNQSLTHTAYDDAVAVHGRLNDSQITKALLNGEFLFELHNGKVVVEQDINTFTSFSP        334
A0A0K2CNL4  A0A0K2CNL4_9CAUD    275    GANANQSLTHTAYDDAVAVHGRLNDSQITKALLNGEFLFELHNGKVVVEQDINTFTSFNP        334
                                       .: .: **:         ..   ..::*  ** .* ::   ...  ::*. :**:*::.

A0A518VEB0  A0A518VEB0_BRELA    347    AEREDFGKIRVSMTLDQIVNDIT-SVGKKYKGKLDNNDIGGATFVGAVKTYLEVREAQGA        405
S5MUG6      S5MUG6_9CAUD        335    DKRKHFSKNRVVRTINGITKDWKLAFDEQYLGKGDNDADGRNLYKKECIKISEQYQAMGA        394
S5MNC1      S5MNC1_9CAUD        335    DKRKHFSKNRVVRTINGITKDWKLAFDEQYLGKGDNDADGRNLYKKECIKISEQYQAMGA        394
S5MCF5      S5MCF5_9CAUD        335    DKRKHFSKNRVVRTINGITKDWKLAFDEQYLGKGDNDADGRNLYKKECIKISEQYQAMGA        394
S5MBG7      S5MBG7_9CAUD        335    DKRKHFSKNRVVRTINGITKDWKLAFDEQYLGKGDNDADGRNLYKKECIKISEQYQAMGA        394
A0A0K2FLW7  A0A0K2FLW7_9CAUD    335    DKRKHFSKNRVVRTINGITKDWKLAFDEQYLGKGDNDADGRNLYKKECIKISEQYQAMGA        394
A0A0K2CNL4  A0A0K2CNL4_9CAUD    335    DKRKHFSKNRVVRTINGITKDWKLAFDEQYLGKYDNDADGRNLYKKECIKIVEEYQAMGA        394
                                        :*:.*.* **  *:: *.:* . :..::* ** **:  *   :     .  *  :* **

A0A518VEB0  A0A518VEB0_BRELA    406    IDQGWIFEDKKN---GIGDKRGFRLAAKPLDAIEIFDIEWEVL---                      445
S5MUG6      S5MUG6_9CAUD        395    IQN---FDAQKDIAVLPGEDSDSLLTEGYIQAVDSMEKNYLKAVAR                      437
S5MNC1      S5MNC1_9CAUD        395    IQN---FDAQKDIIVSPGNDSDSLLTEGYIQPVDAMEKNYLKAVVR                      437
S5MCF5      S5MCF5_9CAUD        395    IQN---FDAQKDIAVLPGEDSDSLLTEGYIQAVDSMEKNYLKAVAR                      437
S5MBG7      S5MBG7_9CAUD        395    IQN---FDAQKDIIVSPGNDSDSLLTEGYIQPVDAMEKNYLKAVVR                      437
A0A0K2FLW7  A0A0K2FLW7_9CAUD    395    IQN---FDAQKDIIVSPGNDSDSLLTEGYIQPVDAMEKNYLKAVVR                      437
A0A0K2CNL4  A0A0K2CNL4_9CAUD    395    IQN---FDAQKDIIVSPGNDSDSLLTEGYIQPVDAMEKNYLKAVVR                      437
                                       *::   *: :*:     *:. .  *:   :: :: :: ::
```

***Highlighted dark grey color amino acids denote similarity among the aligned phage tail-sheath like proteins. (Refer to SM Tables S10 & S11)**

Table S10. Distance matrices of identified *Bl* 1821L and *Bl* 1951 putative phage tail-sheath protein (A0A518VEB0) with the phage tail-sheath proteins of different *Bl* phages .

Uniprot Accession # (https://www.uniprot.org)	Uniprot Accession # (https://www.uniprot.org)						
	**S5MUG6**	**S5MNC1**	**S5MCF5**	**S5MBG7**	**A0A518VEB0**	**A0A0K2FLW7**	**A0A0K2CNL4**
**S5MUG6**		0.01	0	0.01	1.56	0.01	0.03
**S5MNC1**	0.01		0.01	0	1.51	0	0.01
**S5MCF5**	0	0.01		0.01	1.56	0.01	0.03
**S5MBG7**	0.01	0	0.01		1.51	0	0.01
**A0A518VEB0**	1.56	1.51	1.56	1.51		1.51	1.52
**A0A0K2FLW7**	0.01	0	0.01	0	1.51		0.01
**A0A0K2CNL4**	0.03	0.01	0.03	0.01	1.52	0.01	

Table S11. Amino acids alignment (%) of identified *Bl* 1821L and *Bl* 1951 putative phage tail-sheath protein (A0A518VEB0) with the phage tail-sheath proteins of different *Bl* phages.

Uniprot Accession # (https://www.uniprot.org)	Uniprot Accession # (https://www.uniprot.org)						
	S5MUG6	S5MNC1	S5MCF5	S5MBG7	A0A518VEB0	A0A0K2FLW7	A0A0K2CNL4
**S5MUG6**		98.6	100	98.6	21.9	98.6	97.5
**S5MNC1**	98.6		98.6	100	21.7	100	98.9
**S5MCF5**	100	98.6		98.6	21.9	98.6	97.5
**S5MBG7**	98.6	100	98.6		21.7	100	98.9
**A0A518VEB0**	21.9	21.7	21.9	21.8		21.7	21.7
**A0A0K2FLW7**	98.6	100	98.6	100	21.7		98.7
**A0A0K2CNL4**	97.5	98.9	97.5	98.9	21.7	98.9	

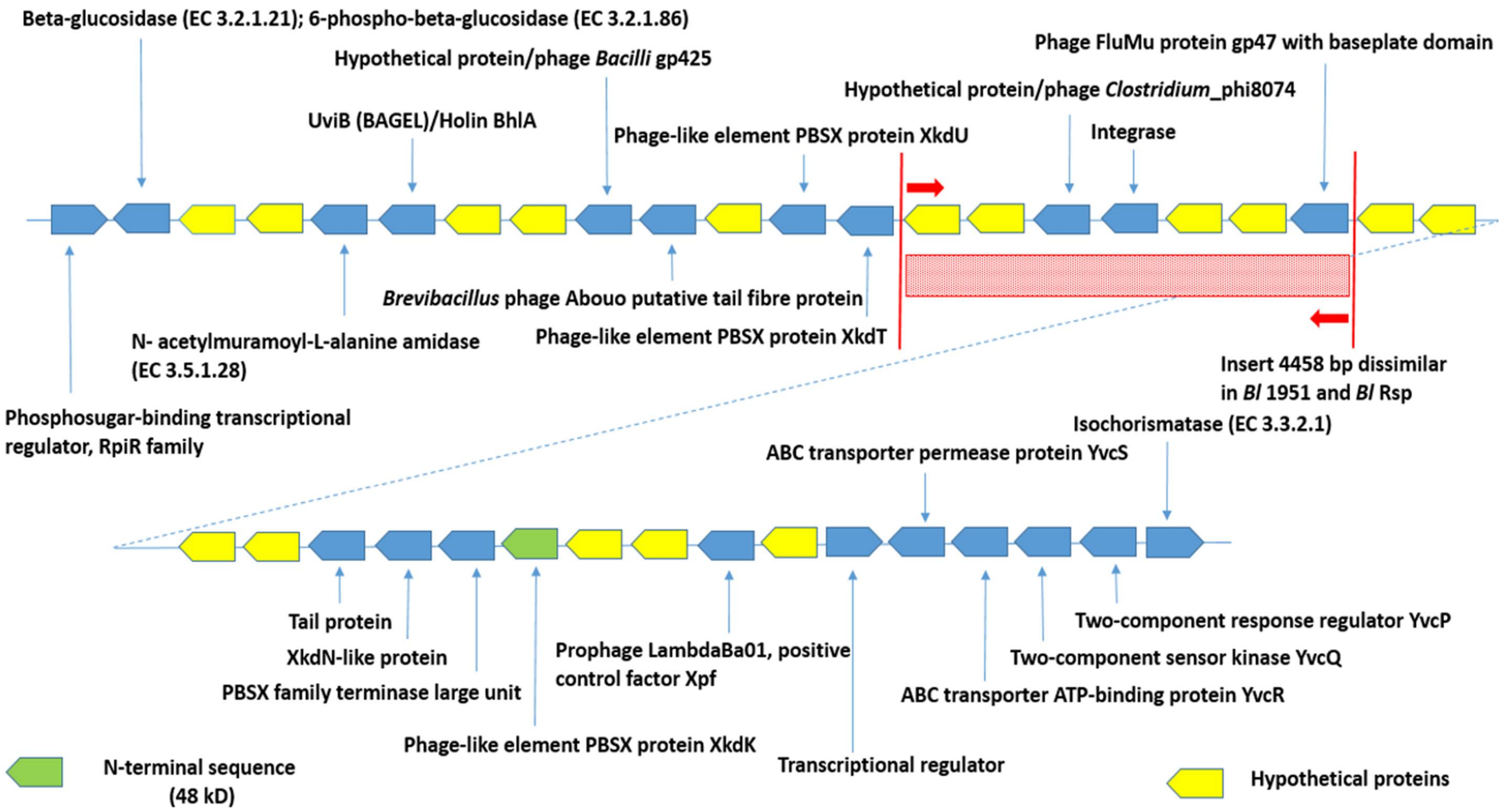

Figure S13. Schematic of the genomic architecture of *Bl* 1821L PBSX-like region encoding the identified gene *xkdK*. The red shaded box denotes *Bl* 1951 region absent in *Bl* 1821L.

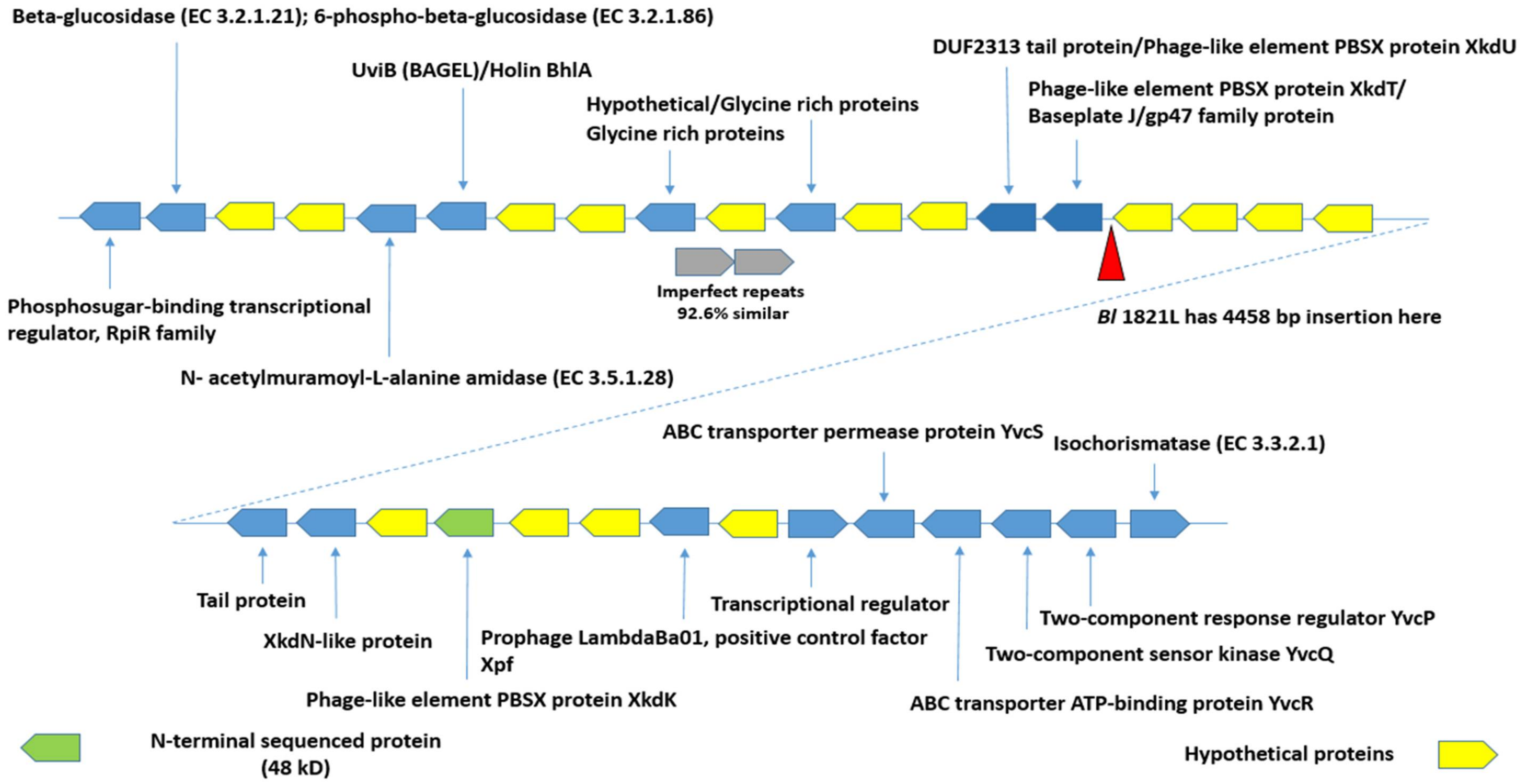

Figure S14. Genomic architecture of *Bl* 1951 PBSX-like region encoding the identified gene *xkdK.* The filled red wedge points to the integration of the red shaded region of *Bl* 1821L (Figure 13) in the *Bl* 1951 genome.

Table S12. Protein orthologues of *Bl* 1821L UviB (BAGEL)/Holin BhlA protein in Uniprot.

Accession	Homologous protein	Organism	% identity
A0A075R9K7	Uncharacterised protein	*Brevibacillus laterosporus* LMG 15441	100
S5M5U3	Bacteriocin UviB	*Brevibacillus* phage Emery	92.0
S5M6A1	Uncharacterised protein	*Brevibacillus* phage Davies	90.8
S5MNE1	Uncharacterised protein	*Brevibacillus* phage Jimmer 1	90.8
A0A0K2CND1	Uncharacterised protein	*Brevibacillus* phage Osiris 1	90.8

Figure S15. Amino acids alignment of identified *Bl* 1821L and *Bl* 1951 phage-like element PBSX protein XkdK (A0A518VEB0) with the similar proteins of the defective prophages PBSZ, PBSX, and PBP180 using programme CLUSTALO.

```
P54331       XKDK_BACSU            1   MNGGTFTTGKEKERAGIYFNFKTTAQERVSLSERGTVALPVASSWGEAKTFVSISSVEDL    60
A0A518VEB0   A0A518VEB0_BRELA      1   -------MTIQRERPGVNVELKAKAQERV-LPKSGVVLVPYLAEWGAPDQVITMKGYEER    52
R4JQA6       R4JQA6_9CAUD          1   MNGGTFTPGTEKKRPGIYFNFKTTAEQRITLGERGTVALPLVMSWGEPKTFISVSDMEDL    60
E0U1S9       E0U1S9_BACPZ          1   MNGGTFTTGKEKERAGIYFNFKTTAQERVSLGERGTVALPVASSWGEAKTFVSISSVEDL    60
                                                 :::* *: .::*:.*::*: * : *.* :*   .**  . .:::.. *: 

P54331       XKDK_BACSU           61   NKKVGLSIDDPSLLLLREAKKNAKTVLMYRLTEGVR--ASADIAEGVKATAVYGGTKGND   118
A0A518VEB0   A0A518VEB0_BRELA     53   V---AETFGH--IGILELAAEGGATVLGYRMTSGNGVAASYTQSDSFTLEARYPGLVGND   107
R4JQA6       R4JQA6_9CAUD         61   NKKVGLNIDDKSLLLFREAKKKAQTVLLYRLNEGEP--AKAEIAENFVVTANYGGQKGNE   118
E0U1S9       E0U1S9_BACPZ         61   NKKVGLSIDDPSLLLLREAKKNAKTVLMYRLTEGVR--ASADIAEGVKATAVYGGTKGND   118
                                           . .:..  : ::. * : . *** **:..*    *.   ::..   * * *  **:

P54331       XKDK_BACSU          119   IIIRINQNVLDANSFDVTTYMDESEVDKQTVKKAEELT----ANGYVTFTGTGDLSSTIP   174
A0A518VEB0   A0A518VEB0_BRELA    108   LQISIKESTVELGKKELQVK-GPIKTEKFSFANVDELVTKAEQSIYIKVKKLGDKVA---   163
R4JQA6       R4JQA6_9CAUD        119   ITIQVAENVLDSTKRDVITYLGTDIVDKQVVKDVKDLV----KNKYVQFSGEGEAVI---   171
E0U1S9       E0U1S9_BACPZ        119   IIIRINPNVLDAASFDVTTYMDESEVDKQTVKKAEELT----ANGYVTFTGTGDLSSTIP   174
                                       : * :  ..::  . ::  .  .   .:*  . ...:*.     . *: ..  *:

P54331       XKDK_BACSU          175   LTGSEGDTAAETLNASAGIRLSGGTDKAPV--NSDYTDFLAAAETESFDVIALPVAEGDQ   232
A0A518VEB0   A0A518VEB0_BRELA    164   -------------EETMMISLEGGTSGITSLTANDFTTLFNSISGTDFDAMYLPSADVAI   210
R4JQA6       R4JQA6_9CAUD        172   ---------------TAGAALSGGKNGVAS--VADYTAFLEAAETEYFDVIALPVDNSEQ   214
E0U1S9       E0U1S9_BACPZ        175   LTGSEGDTSAETLNASAGIRLSGGTDKAPV--NSDYTDFLAAAETENFDVIVLPVAEGDQ   232
                                                      :    *.**..        *:* :: : .   **.: **  :

P54331       XKDK_BACSU          233   LKAT--F------------AAFIKRLRDGQGQKVQGVTANYAGDYEGIINVTEGVLLEDG   278
A0A518VEB0   A0A518VEB0_BRELA    211   QAAAKQFMSDRELFSKKRSTLVIGGLKDKDNNMNEHVQRSVANNSRRVVNCAIAGQHVNG   270
R4JQA6       R4JQA6_9CAUD        215   LKAT--F------------ASFIERLRDKQGRKVQGVVANYAADQEGIINVTSGVVLEDG   260
E0U1S9       E0U1S9_BACPZ        233   LKAT--F------------AAFIKRLRDGQGQKVQGVTANYAGDYEGIINVTEGVLLEDG   278
                                         *:  *            : .*  *:* :..  : *  . * : . ::* : .    :*
```

```
P54331      XKDK_BACSU       279   TEVTPDKATAWVAGASAGATFNQSLT--FVEYEGAVDVLHRLDHDTIVERLGKGEFLFTF   336
A0A518VEB0  A0A518VEB0_BRELA 271   KTYGSLEWAAWLAGMIAATPAHISLSAQLVPLKKA---EKDWGHTDIQAALNSGTLIAVR   327
R4JQA6      R4JQA6_9CAUD     261   TELTPAQTTAWVAGASAGANFNQSLT--FVEYEGAVDTLERLDNDQVEYRLSQGEFLFTF   318
E0U1S9      E0U1S9_BACPZ     279   TEVTPDKATAWVAGASAGATFNQSLT--FVEYEGAVDVLHRLDHDTIVERLGKGEFLFTF   336
                                   .     : :**:**  *.:  : **:  :*  : *    . .:   :   *..* :: .

P54331      XKDK_BACSU       337   DARDKSVSVEKDINSLVTFTAEKNKKFAKNKIVRVLDAVNNDLTRELKALIKSRKGSGSD   396
A0A518VEB0  A0A518VEB0_BRELA 328   D--GDVYLIESAVNTLTTLKAAEREDFGKIRVSMTLDQIVNDITSVGKKYKGK--LDNND   383
R4JQA6      R4JQA6_9CAUD     319   DARDRTVSVEKDINSLTSFTVEKNQQMAKNKIIRVLDAINNDLTFELKNLIKLRKANGND   378
E0U1S9      E0U1S9_BACPZ     337   DARDKSVSVEKDINSLVTFTAEKNKKFAKNKIVRVLDAVNNDLTRELKALIKSRKGSGSD   396
                                   *  .    :*. :*:*.::.. :.:.:.* ::  .** : **:*   *        ...*

P54331      XKDK_BACSU       397   IPASEDGLQYVKTMITQYMTTLQDAGGIT-GFDSDEDITISMNEDRDGFLIDLAVQPVDA   455
A0A518VEB0  A0A518VEB0_BRELA 384   I----GGATFVG-AVKTYLEVREAQGAIDQGWI-FEDKKNGI-GDKRG--FRLAAKPLDA   434
R4JQA6      R4JQA6_9CAUD     379   IPASDDGVQLVKTLITQYLTQLQDGSGIT-GFNSETDIVIGLNEDRDGFIIDLAVQPVDA   437
E0U1S9      E0U1S9_BACPZ     397   IPASEDGLQYVKTMITQYMTTLQDAGGIT-GFDSDEDITISMNEDRDGFLIDLAVQPVDA   455
                                   *    .*   *   :. *:   :  ..*  *:    *   .:  *: *  : **.:*:**

P54331      XKDK_BACSU       456   AEKFYFNVEVN   466
A0A518VEB0  A0A518VEB0_BRELA 435   IEIFDIEWEVL   445
R4JQA6      R4JQA6_9CAUD     438   AEKFYFNVEVK   448
E0U1S9      E0U1S9_BACPZ     456   AEKFYFNVEVN   466
                                    * * :: **
```

***Highlighted dark grey color amino acids denote similarity among the aligned phage-like element PBSX protein XkdK.**
(Refer to SM Tables S13 & S14)

Table S13. Distance matrices of identified *Bl* 1821L and *Bl* 1951 phage-like element PBSX protein XkdK (A0A518VEB0) with the similar proteins of the defective prophages PBSZ, PBSX, and PBP180.

Uniprot Accession # (https://www.uniprot.org)	Uniprot Accession # (https://www.uniprot.org)			
	**P54331**	**A0A518VEB0**	**R4JQA6**	**E0U1S9**
**P54331**		1.45	0.37	0.01
**A0A518VEB0**	1.45		1.40	1.47
**R4JQA6**	0.37	1.40		0.37
**E0U1S9**	0.01	1.47	0.37	

(Refer to SM Figure S15)

Table S14. Amino acids alignment (%) of identified *Bl* 1821L and *Bl* 1951 phage-like element PBSX protein XkdK (A0A518VEB0) with the similar proteins of the defective prophages PBSZ, PBSX, and PBP180.

Uniprot Accession # (https://www.uniprot.org)	Uniprot Accession # (https://www.uniprot.org)			
	**P54331**	**A0A518VEB0**	**R4JQA6**	**E0U1S9**
**P54331**		22.9	66.5	98.7
**A0A518VEB0**	22.9		21.6	22.9
**R4JQA6**	66.5	21.6		66.5
**E0U1S9**	98.7	22.9	66.5	

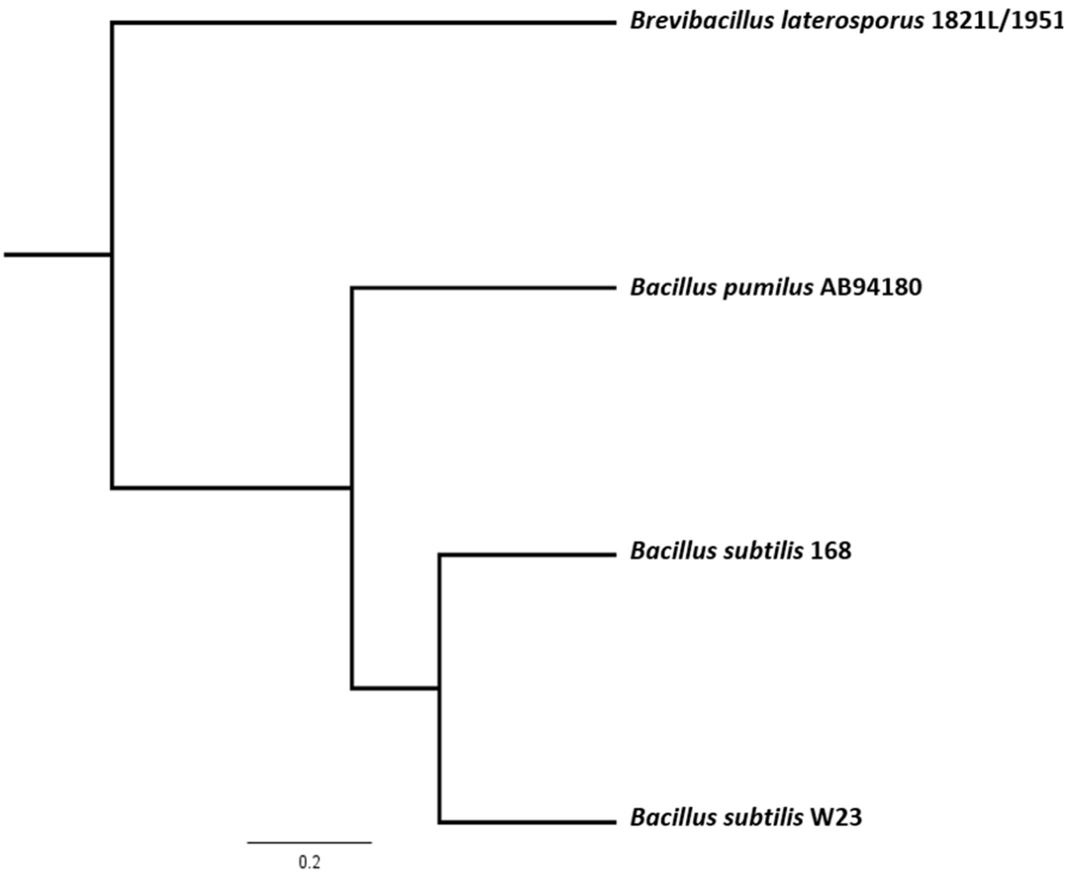

Figure S16. Dendrogram showing alignment of the identified phage-like element PBSX protein XkdK (A0A518VEB0) of *Bl* 1821L and *Bl* 1951 with similar proteins of defective prophages of *Bs* W23 (E0U1S9), *Bs* 168 (P54331), and *Bacillus* phage PBP180 (R4JQA6). Key is 0.2 nt substitutions per site.

Figure S17. Amino acids alignment of tail protein (A0A518VEA0) encoded in PBSX-like region of *Bl* 1821L and *Bl* 1951 with the similar proteins of the defective prophages PBSZ, PBSX, and PBP180 using programme CLUSTALO.

```
A0A518VEA0 A0A518VEA0_BRELA    1  ---------MNNDMVVELLSVQKEMYKVRQGMMEWKRDA--VSL--QVAWGQMGRDFLRE    47
E0U1T3     E0U1T3_BACPZ        1  MAKLTARFDMEDRVS-------KKLRKVQNGFRALEKYRKMVQRKSAIDIRKDSKTILRT    53
R4JJ03     R4JJ03_9CAUD        1  ------------------------------------------------------------     0
P54334     XKDO_BACSU          1  MAKLTARFDLEDKVS-------KKLKRIHKGFQEVEKKVKTINRQIKISIRAEDQAFY--    51

A0A518VEA0 A0A518VEA0_BRELA   48  TDKMERRIK---------------------ELHSQLKRLGT-------------------    67
E0U1T3     E0U1T3_BACPZ       54  IDRIQKSLKKKLGSQMISISAEDNASAVIQQVQTQLQGLPASVSINIGASDQATERFERL   113
R4JJ03     R4JJ03_9CAUD        1  ------------------------------------------------------------     0
P54334     XKDO_BACSU         52  ------------------------------------------------------------    51

A0A518VEA0 A0A518VEA0_BRELA   68  ------------------------------------------------------------    67
E0U1T3     E0U1T3_BACPZ      114  RELVAGFKGFTIVLSAEDQVSPAVQKIQRYMETALKNGYSVTIRVIDHVMKTVGRISAGI   173
R4JJ03     R4JJ03_9CAUD        1  ------------------------------------------------------------     0
P54334     XKDO_BACSU         52  ------------------------------------------------------------    51

A0A518VEA0 A0A518VEA0_BRELA   68  ----SVQAKLRVTIDDQATQKISQ-------MRSQISQSLVISGGGSSGGSKASVMDPMG   116
E0U1T3     E0U1T3_BACPZ      174  DALTGKDNKLELAINDKVSDKLDSLQKRIDSMRSSGSPDKAAPSAGGNTGDIASMFDPET   233
R4JJ03     R4JJ03_9CAUD        1  ------------------------------------------------------------     0
P54334     XKDO_BACSU         52  ------------------------------------------------------------    51

A0A518VEA0 A0A518VEA0_BRELA  117  DY-----LINSYNDAIPDSQAAAKE----------RGLFMARGKT---ELEGQ----ELD   154
E0U1T3     E0U1T3_BACPZ      234  ILTALDKFASSFMEKVDEIATKFSPETILTELDKFTTSFMNKVDAIATKFSPETILTQLD   293
R4JJ03     R4JJ03_9CAUD        1  ------------------------------------------------------------     0
P54334     XKDO_BACSU         52  ------------------------------------------------------------    51

A0A518VEA0 A0A518VEA0_BRELA  155  -----------------------------------------------RSVGRMTQINPEM   167
E0U1T3     E0U1T3_BACPZ      294  KFTTSFMSKVDAIATKFSPETILTQLDKFTTSFMSKVDAIATKFSPETILGQLDKFTTSF   353
R4JJ03     R4JJ03_9CAUD        1  ------------------------------------------------------------     0
P54334     XKDO_BACSU         52  ------------------------------------------------------------    51
```

```
A0A518VEA0 A0A518VEA0_BRELA 168 --------MKAEATAIYNRSDEVNPTN----KAEYAEFAAKLSMSTGFTSDQSLKMMALL  215
E0U1T3     E0U1T3_BACPZ     354 MSKVDAIATKFSPETILEQLDKFTTSFMGKVDAIAAKFSPET-ILSQL-DKFTTSFMNKV 411
R4JJ03     R4JJ03_9CAUD       1 --------MKISLEMDEKKLRKFNLSM---------------------------------  19
P54334     XKDO_BACSU        52 ------------------RLRKI-------NDYIILKFAKSLEIKVVL-DDQ---VTSKL  82
                                                  :  :.

A0A518VEA0 A0A518VEA0_BRELA 216 RDSTGVNDPERLANSLQYMST-NMK-------DFSDDFVSSMIKNTSQLGLLMDTPEKMA 267
E0U1T3     E0U1T3_BACPZ     412 DAIATKFSPETLLKQLDKFTGSFMKKVDEIASKFSPEAIFKQLD--KFTGSFMKKVDDVV 469
R4JJ03     R4JJ03_9CAUD      20 ---------------RRFSVLSMKLYQ---------GIYRDLR--SLANQLNMIPKQMV  52
P54334     XKDO_BACSU        83 DAIERKL------KRLPKETKLTVSLVDRTAAAYKK--IKKMLS--NQKLSFLLIPDDKV 132
                                                   :   :.             :   :   .   :   .. .

A0A518VEA0 A0A518VEA0_BRELA 268 MLVGEIGN---------M---GIPSNGLPLEA-----------LKDLALKMSTQGEMSKV 304
E0U1T3     E0U1T3_BACPZ     470 S--------------------KFSPEV-IFKQLDKFTGSFMKKVDDVVSKFSP-EAIFKQ 507
R4JJ03     R4JJ03_9CAUD      53 ISIQAKGLDVIKSSIDRLKQSGTSPILLTFKLNDQLSGKMSS-IK---------KSILQL 102
P54334     XKDO_BACSU       133 T-------------------ST---------VKRIIGYIKKN----------------  146

A0A518VEA0 A0A518VEA0_BRELA 305 LQRGYEADGKSPEEAKRLATIESKEVTQLLHSDNKSDNQMAMGRIFMNVASIKDDNVRQE 364
E0U1T3     E0U1T3_BACPZ     508 LDKFADSFMKKVDDIVS--------------------KFSPATIFNELDKFADSFM-KK 545
R4JJ03     R4JJ03_9CAUD     103 MNRTYYMRLNMVDQATT---------------------AI------QRIKKT--------  127
P54334     XKDO_BACSU       147 LKNGYTVKLKVIDEITK---------------------TV------NR------------  167
                                :..      :  ::                       .        .

A0A518VEA0 A0A518VEA0_BRELA 365 MLNEVGSGSGK----ELLQYLEPLIESAGNI----SA---------------------G 394
E0U1T3     E0U1T3_BACPZ     546 VDDVVSKFSPETIFNELDKFTDSFMKKVDDIVSKFSPDAILTKAEDFLTNIVDKISEKFN 605
R4JJ03     R4JJ03_9CAUD     128 ----------------L----KSLTMAKHEI---------RVSVQDNAKSKLKKRDQAES 158
P54334     XKDO_BACSU       168 ------------LTALLRKFEKTYYVKVV----------------VKTKISDKINKKES 198
                                                *    .                                   .

A0A518VEA0 A0A518VEA0_BRELA 395 LVVNKVADDEA-----EKSYQAAKDQNPWFEYMQAQNEVRLAMTELAATVAKDL------ 443
E0U1T3     E0U1T3_BACPZ     606 FLNPDKI---------------A--------------------------------- 613
R4JJ03     R4JJ03_9CAUD     159 VVKEKKIKKKP---NATAATKTKENKQSWLGNLGNKALKE--IEKYAGDVSDKLKEKLSP 213
P54334     XKDO_BACSU       199 LINKETSKKESDKSSDKGSDKNSDQKPGWVGKIK---------------------GWL 235
                                .:  .

A0A518VEA0 A0A518VEA0_BRELA 444 -----APGFKILSNGIANVVQSFNKL---------P-QMARLAIEGVVIAFLAKKAKKSL 488
E0U1T3     E0U1T3_BACPZ     614 -----NKAEKFFDNIVSKIAKKFEKFSPDKIIEK-VGEFFEKIIKGIAEKLVNLDFGGLL 667
R4JJ03     R4JJ03_9CAUD     214 RKFWDEKALPWVETKIDEYKQEVIGRISEKIKIN-PEEY--------LDKWFNKGLDLIL 264
P54334     XKDO_BACSU       236 KDWWTKSAWPWIKDTLNEIGKAFLDRFKERLIEKLKNDVFDKIID------------KIL 283
                                     .   ..  : :   : .              :                    *
```

```
A0A518VEA0 A0A518VEA0_BRELA   489  QGEQGDSKNI--------TIKKI-----DVKPKITIKNKVN--VTLQDKKKVICQCSCSG   533
E0U1T3     E0U1T3_BACPZ       668  GGKSSGSKGKQKASKANTNLSTSNNSNRTKKPSMNQKASGSKSKKSGGKWGGACGCCCAG   727
R4JJ03     R4JJ03_9CAUD       265  GPKNQSSENNTNSSAQNQTTETP------TNPTPNTQ--QNNRNGKGLFRSSCCPCCARG   316
P54334     XKDO_BACSU         284  GPQKKDDSGEN--------------S-DTDKKVLN--------RNTGG--NCCCDCCEGG   318
                                     :. ....                     :   .                  * *.  *

A0A518VEA0 A0A518VEA0_BRELA   534  DSDAGGQQRKKRKGSKGKRKGKRSQKQRGPSKNGGKNSGGASKQNRNNPSNPSNQKKPNK   593
E0U1T3     E0U1T3_BACPZ       728  ISTGKSKKVKNRNGSSNKGN-------------------------KSNPVNT-----P--   755
R4JJ03     R4JJ03_9CAUD       317  LSRRGSNRTRNRNGRSQRQP-------------------------SNPTATA-----RTE   346
P54334     XKDO_BACSU         319  ISPGTKNKKTYRKKKSPTKA-------------------------PNRTLKV-----PGE   348
                                    *    ::   *:  .                              .   .

A0A518VEA0 A0A518VEA0_BRELA   594  PNSQPKST---KPLNRKGNIPTPAPSKKGKLATGFGKLKDIGGKAFEGIKNFGGSTWDGL   650
E0U1T3     E0U1T3_BACPZ       756  -----------KSAKA---------------------A--KGGS---------GKGFSGL   772
R4JJ03     R4JJ03_9CAUD       347  RYRRPQ-GRLGKLKTSAGKIFEK-------VPSGLKKTTGIVAS---------VAGLAGL   389
P54334     XKDO_BACSU         349  VIRRRQSGTPAESAPR---------------------T--SGSK---------LKNWLGG   376
                                              :                              ..              *

A0A518VEA0 A0A518VEA0_BRELA   651  -KNLGGK-G----LGGAKSFGKGLLKKIPFVGEAMGLASLATSDNKPM--ELLKLG----   698
E0U1T3     E0U1T3_BACPZ       773  LKTLGESKG----LKGGLKGLKGAAKGIPGLGEILSLTDLAGINKDNAGEKVGSAGGGLA   828
R4JJ03     R4JJ03_9CAUD       390  MKGSGGLSGLGDVLKNIGKGSSQLLKRVPILGNLLSATDLIGTTKENVGEKVGGFSGGLA   449
P54334     XKDO_BACSU         377  LKSLGKDSS----KL--GKAFRGMGKAVPYVGSALAVFDLAGINKDNAGEKVGSIGGRIA   430
                                    *  *   .         .      * :* :*. :.  .*    :.    ::   .

A0A518VEA0 A0A518VEA0_BRELA   699  GSAGMKAVGTMIGATVGSIVPGLGTAVGGVVGGYLGSVGGDFLMDKL----PDWFGWGKE   754
E0U1T3     E0U1T3_BACPZ       829  GAAAG--------AAIGSVVPGVGTAIGGVVGG----IAGTFGGESLGK----AIDAGA-   871
R4JJ03     R4JJ03_9CAUD       450  GGISGAAI---------GQTLIPI-PFVGAAVGGVAGSIAGTLGGSKL----GELFDTSKL   496
P54334     XKDO_BACSU         431  GAEAGATAGAEAGATIGSFVGPEGTAIGGAIGGIGGGLVGGIGGEEAGKNIGKMFDTSKL   490
                                   *. .            *. :      :*..:**    : * :  ..        :. .

A0A518VEA0 A0A518VEA0_BRELA   755  KPQPPPAPV------------P-----PPAPVPPAQPPIPTDFDASQIKPMRRKPQNTLA   797
E0U1T3     E0U1T3_BACPZ       872  -------------LNDTWNSITEGAQNAWSAIQGTWGTVSTWFTDNV-----WTPVSSAV   913
R4JJ03     R4JJ03_9CAUD       497  KENISNTLFSEDWWSEKWGSVKTSAEKSLGNLSEKWEDIKTTASNTLFNSEWWAEQAGYI   556
P54334     XKDO_BACSU         491  KESISNSLFNKEWWSGKWNSISDTANGAITWIEDTWSKVSTWFDQYV-----FTPIYNVA   545
                                                                  :      : *

A0A518VEA0 A0A518VEA0_BRELA   798  ITPAPPVKPVATNAVNKKESAQNLSVTVSTMPITLHADGVLQDVVGMIRLLKDPSVTNEV   857
E0U1T3     E0U1T3_BACPZ       914  MGV---ATSIWSNIVNAWTTIQTIFSTV----STWFMDNVWTPVSSAVV---------GV   957
R4JJ03     R4JJ03_9CAUD       557  TGA---LE------------------------STVFNGEWWSEKWDAIK---------DW   580
P54334     XKDO_BACSU         546  VPV---INFIVGLFAYAWDGILAVWNIV----YPWFKDNVWDPISGAVT---------NV   589
                                                                       . .       . :
```

```
A0A518VEA0  A0A518VEA0_BRELA   858  KRIIETAFVNALETRGGKA-----------------------------------------     876
E0U1T3      E0U1T3_BACPZ       958  ATAIWSKIVNAWTTIKNVFSTVASWFMSNVWGPVKSAVIGAATTIWDKMTGAWTKIKSV-    1016
R4JJ03      R4JJ03_9CAUD       581  TQEKWDGAVEIWNSITKKLGE--TVFNGEWWGEKWDSVKKWTKEKWDGAVNIWNSIKGKI     638
P54334      XKDO_BACSU         590  ATTIWTGLCNAWTVISGIWSTVSTWFMDNVWNPVSEAVTTAATWIWSKINDAWTIISNI-     648
                                             :

A0A518VEA0  A0A518VEA0_BRELA   877  ------------------------------------------------------------     876
E0U1T3      E0U1T3_BACPZ      1017  -------------FSTVSGWFMDIVWNPVKNTVLDVGKGISDAFKKAIDTVKNIWKGLSG    1063
R4JJ03      R4JJ03_9CAUD       639  RETVFNKDWWGEKWDDVKNWSE-NKWEQSKTIWS-AAKATASSTLFNKNWWTNKWNDVKS     696
P54334      XKDO_BACSU         649  -------------WTGVSNWFYDNVWNPIVKKVEDIKTSIKEKFDDARKFFEDAWDGIET     695

A0A518VEA0  A0A518VEA0_BRELA   877  ------------------------------------------------------------     876
E0U1T3      E0U1T3_BACPZ      1064  WFKKNIQEPLTKVGEAISDAFSKAFGWVKQIWDKAGGVASKVINFVTGGGD---------    1114
R4JJ03      R4JJ03_9CAUD       697  WGKNILG----------------------DTWDLIKSKGAEVIGRH----------IV--     722
P54334      XKDO_BACSU         696  WFNEHVKDPLVKVSEKIAEK----FQWLFDLKDSVGGFVAKVIHRGEEVTHLKKKPIKKD     751

A0A518VEA0  A0A518VEA0_BRELA   877  ------------------------------------------------------------     876
E0U1T3      E0U1T3_BACPZ      1115  -------------------------PNKGKDPDKKATGGYITKPTISWIGEAGKEFVIPV    1149
R4JJ03      R4JJ03_9CAUD       723  -----------------KFEKGREKGRKDFKPDQKATGGYITQPTLSWIGEAGNEFVIPT     765
P54334      XKDO_BACSU         752  SKEKKTSSKPTTNNIFAQSPSNNNMTVFQQALDQNATGGYITKPTISWIGEAGKEFVIPV     811

A0A518VEA0  A0A518VEA0_BRELA   877  ------------------------------------------------------------     876
E0U1T3      E0U1T3_BACPZ      1150  DNNRGRGKMLLSQAASKLGMQVVDDMG-AASSSSGNPVSVSGGAASSPLSGSASP-SMDT    1207
R4JJ03      R4JJ03_9CAUD       766  QNNRGRGKMLLAQAASHLGMSVVPSSSGAASPVSSSPAPITPNAPAAPTASAGSIGGTVS     825
P54334      XKDO_BACSU         812  DNNRGRGKMLLSQAASKLGMQVVDDMG-AASSSGGSPASVSGGAAVSPLSGTASP-VMNT     869

A0A518VEA0  A0A518VEA0_BRELA   877  ------------------------------------------------------------     876
E0U1T3      E0U1T3_BACPZ      1208  ANLTGQASTLGQQFSEGFGKGISNQPVKMEDWKKKNINTPFTQMISSSQNYGKQMVSGYA    1267
R4JJ03      R4JJ03_9CAUD       826  MNGNIQSASIGEQFNHDFEQGLNQKVVSLDQWKQKNIQQPFNQMTSDSGKYGQQTVSAFA     885
P54334      XKDO_BACSU         870  ANLTGQASTLGQQFSEGFGKGISDQPVKMEDWKKKNINTPFTQMISASPNYGKQMVSGYA     929

A0A518VEA0  A0A518VEA0_BRELA   877  ------------------------------------------------------------     876
E0U1T3      E0U1T3_BACPZ      1268  KGQNGTATGTDGFLQSKVKTPFQATVNKSSSWGTETVKGFAQGQNSTQTGTAQYVSTHVD    1327
R4JJ03      R4JJ03_9CAUD       886  TGQQMTPTGTDSFLQSRVKAPYQQVMTASPTWGSGTVSGFATGQNATSIGTSQYVDQHVK     945
P54334      XKDO_BACSU         930  KGQSGTATGTDGFLQSKVKTPFQATVSKSSSWGTGTVKGFAQGQNSTQTGTAQYVSTHVD     989
```

```
A0A518VEA0 A0A518VEA0_BRELA  877 ------------------------------------------------------------  876
E0U1T3     E0U1T3_BACPZ     1268 KGQNGTATGTDGFLQSKVKTPFQATVNKSSSWGTETVKGFAQGQNSTQTGTAQYVSTHVD 1327
R4JJ03     R4JJ03_9CAUD      886 TGQQMTPTGTDSFLQSRVKAPYQQVMTASPTWGSGTVSGFATGQNATSIGTSQYVDQHVK  945
P54334     XKDO_BACSU        930 KGQSGTATGTDGFLQSKVKTPFQATVSKSSSWGTGTVKGFAQGQNSTQTGTAQYVSTHVD  989

A0A518VEA0 A0A518VEA0_BRELA  877 ------------------------------------------------------------  876
E0U1T3     E0U1T3_BACPZ     1328 KPFLRSKDTSNSWGSGMVGNFVTGMNSKSSEVKQAAKDMAKRVEQAFREELDIHSPSRVM 1387
R4JJ03     R4JJ03_9CAUD      946 QPFLQAKQESPGWGSGMIDAFNSGMRSKASEVTQAAKEMAKKVEQAFREELDIHSPSRVM 1005
P54334     XKDO_BACSU        990 KPFLRSKDTSNSWGSGLIGNFVTGMNSKSSEVKQAAKDMAKRVEQAFREELDIHSPSRVM 1049

A0A518VEA0 A0A518VEA0_BRELA  877 ------------------------------------------------------------  876
E0U1T3     E0U1T3_BACPZ     1388 MSLGRFASIGVVKGLGSVDVKKYAEKQAGSLAAAYSGMGAMSGNVKQWIMAALMATKTPM 1447
R4JJ03     R4JJ03_9CAUD     1006 MSLGKFASIGVVKGLDSVDVKKFAENQAGSLIGAFSGMGASGLSVQQWLMAALMATGTSM 1065
P54334     XKDO_BACSU       1050 MSLGRFASVGVVKGLDSVDVKKYAEKQAGSLAAAYSGMGAVGGNVKQWLMAAIMATKTPL 1109

A0A518VEA0 A0A518VEA0_BRELA  877 ------------------------------------------------------------  876
E0U1T3     E0U1T3_BACPZ     1448 SWLPGLMTIAQHESGGNPNAINLWDSNAKAGHPSQGLMQTIPSTFNDHKAPSMGNIKNPI 1507
R4JJ03     R4JJ03_9CAUD     1066 SWLPGLMTIAQHESNGNPKAINLWDSNAKKGTPSKGLMQTIGPTFHSNKGKGMNDIWNPI 1125
P54334     XKDO_BACSU       1110 SWLSGLMTIAQYESGGNPNSINLWDSNAKAGNPSQGLMQTVPTTFNAHKAPGMGNIRNPI 1169

A0A518VEA0 A0A518VEA0_BRELA  877 ------------------------------------------------------------  876
E0U1T3     E0U1T3_BACPZ     1508 HNAAAAIGYIKSRYGSINNVPGIKSLNHGGPYVGYANGGLITKEQIARVGEGNKREWIIP 1567
R4JJ03     R4JJ03_9CAUD     1126 HNAVAAINYIKGRYGTVFNTPGLRSMRRGGPYKGYANGGLITQEQVARVGEGNKREWIIP 1185
P54334     XKDO_BACSU       1170 HNAAAAIGYIKSRYGSIDNVPGIKSLKRGGPYVGYANGGLITKEQIARVGEGNKREWIIP 1229

A0A518VEA0 A0A518VEA0_BRELA  877 ------------------------------------------------------------  876
E0U1T3     E0U1T3_BACPZ     1568 EERGIRGRYLLQKAAQALGMEVTDPSQSQ-SDLSSGQVSAI-TAGTRQTIHTAGTKEIKI 1625
R4JJ03     R4JJ03_9CAUD     1186 EERGIRGRYLLTQAAKALGMQVYDPSNASAPL-PESQMQQVTSAQSAGNTTTSGNKQITI 1244
P54334     XKDO_BACSU       1230 EERGIRGRYLLQRAAQALGMEVTDPSQSQQSELSSGQVSAV-TSASRPTAAVSGSKEIYI 1288

A0A518VEA0 A0A518VEA0_BRELA  877 --------------------------------------------                  876
E0U1T3     E0U1T3_BACPZ     1626 EFNGDQHFHNEQDADSLVAKIKQALLDELQKDINTGTKGVVAFD                 1669
R4JJ03     R4JJ03_9CAUD     1245 QFNGDQHFHNGQDQQSLVEKIRQMLVDELEVELQTGTKGVVIDG                 1288
P54334     XKDO_BACSU       1289 QFNGDQHFHNGQDAESLAAKIKQALIDELQKDINIGTKGVVAFD                 1332
```

***Highlighted dark grey color amino acids denote similarity among the aligned phage-like element PBSX protein XkdK.**
(Refer to SM Tables S15 & S16)

Table S15. Distance matrices of tail protein (A0A518VEA0) encoded in PBSX-like region of *Bl* 1821L and *Bl* 1951 with the similar proteins of the defective prophages PBSZ, PBSX, and PBP180.

Uniprot Accession # (https://www.uniprot.org)	Uniprot Accession # (https://www.uniprot.org)			
	**P54334**	**E0U1T3**	**R4JJ03**	**A0A518VEA0**
**P54334**		0.56	0.85	0.71
**E0U1T3**	0.56		0.84	1.73
**R4JJ03**	0.85	0.84		1.79
**A0A518VEA0**	0.71	1.73	1.79	

Table S16. Amino acids alignment (%) of tail protein (A0A518VEA0) encoded in PBSX-like region of *Bl* 1821L and *Bl* 1951 with the similar proteins of the defective prophages PBSZ, PBSX, and PBP180.

Uniprot Accession # (https://www.uniprot.org)	Uniprot Accession # (https://www.uniprot.org)			
	**P54334**	**E0U1T3**	**R4JJ03**	**A0A518VEA0**
**P54334**		51	37	0
**E0U1T3**	51.9		37.1	50
**R4JJ03**	37	37.1		0
**A0A518VEA0**	0	50	0	

Figure S18. Amino acids alignment of holin protein (A0A075R9K7) encoded in PBSX-like region of *Bl* 1821L and *Bl* 1951 with the similar protein of the defective prophages PBSZ, PBSX, and PBP180 using programme CLUSTALO.

```
A0A075R9K7 A0A075R9K7_BRELA 1    ----------------------------------MEES---VMNALLQQGP-----FAAL    18
E0U1U5     E0U1U5_BACPZ     1    MKMFDKGTVIRTVLLLVALINQTMLMLGKSPLDIQEEQVNQLADALYSAGSVIFTIGTTL    60
R4JHG4     R4JHG4_9CAUD     1    MKTFDKGTVIRTVLLFIALINQTLVMFGQTVLPISEEQVQTAGEALYVAGSTIFTMVTAV    60
Q99163     XHLB_BACSU       1    MNTFDKGTVIRTVLLLIALINQTMLMLGKSPLDIQEEQVNQLADALYSAGSIAFTIGTTL    60
                                                                   **.     :**   *       :::

A0A075R9K7 A0A075R9K7_BRELA 19   FVWLLFS--TKKEGRDREALLVKQAQTREAKLMEHNERMVIQLERNTSTLQQIERSLSGL    76
E0U1U5     E0U1U5_BACPZ     61   AAWFKNNYVTEKGKKQRD-LLKENNLTK--------------------------------    87
R4JHG4     R4JHG4_9CAUD     61   IAWFKNNYVTYKGQLQKD-ALKQRGLTK--------------------------------    87
Q99163     XHLB_BACSU       61   AAWFKNNYVTEKGKKQRD-LLRDNNLTK--------------------------------    87
                                  .*:  .  * *   :::  *  ..  *:

A0A075R9K7 A0A075R9K7_BRELA 77   EMELQELKEKVE    88
E0U1U5     E0U1U5_BACPZ     88   ------------    87
R4JHG4     R4JHG4_9CAUD     88   ------------    87
Q99163     XHLB_BACSU       88   ------------    87
```

***Highlighted dark grey color amino acids denote similarity among the aligned holin proteins.**

(Refer to SM Figure S19 & Tables S17 and S18)

Table S17. Distance matrices of holin protein (A0A075R9K7) encoded in PBSX-like region of *Bl* 1821L and *Bl* 1951 with the similar proteins of the defective prophages PBSZ, PBSX, and PBP180.

Uniprot Accession # (https://www.uniprot.org)	Uniprot Accession # (https://www.uniprot.org)			
	**A0A075R9K77**	**E0U1U5**	**R4JHG4**	**Q99163**
**A0A075R9K77**		2.41	1.48	1.89
**E0U1U5**	2.41		0.45	0.08
**R4JHG4**	1.48	0.45		0.47
**Q99163**	1.89	0.08	0.47	

Table S18. Amino acids alignment (%) of holin protein (A0A075R9K7) encoded in PBSX-like region of *Bl* 1821L and *Bl* 1951 with the similar proteins of the defective prophages PBSZ, PBSX, and PBP180.

Uniprot Accession # (https://www.uniprot.org)	Uniprot Accession # (https://www.uniprot.org)			
	**A0A075R9K77**	**E0U1U5**	**R4JHG4**	**Q99163**
**A0A075R9K77**		13.6	11.4	13.6
**E0U1U5**	13.6		64.4	89.8
**R4JHG4**	11.4	64.4		61.4
**Q99163**	13.6	89.8	61.6	

Figure S19. **Schematic presentation of amino acids alignment of holin protein (A0A075R9K7) encoded in PBSX-like region of *Bl* 1821L and *Bl* 1951 with the similar proteins of the defective prophages PBSZ, PBSX, and PBP180 using programme Geneious basic (A) and CLUSTALO (B).**

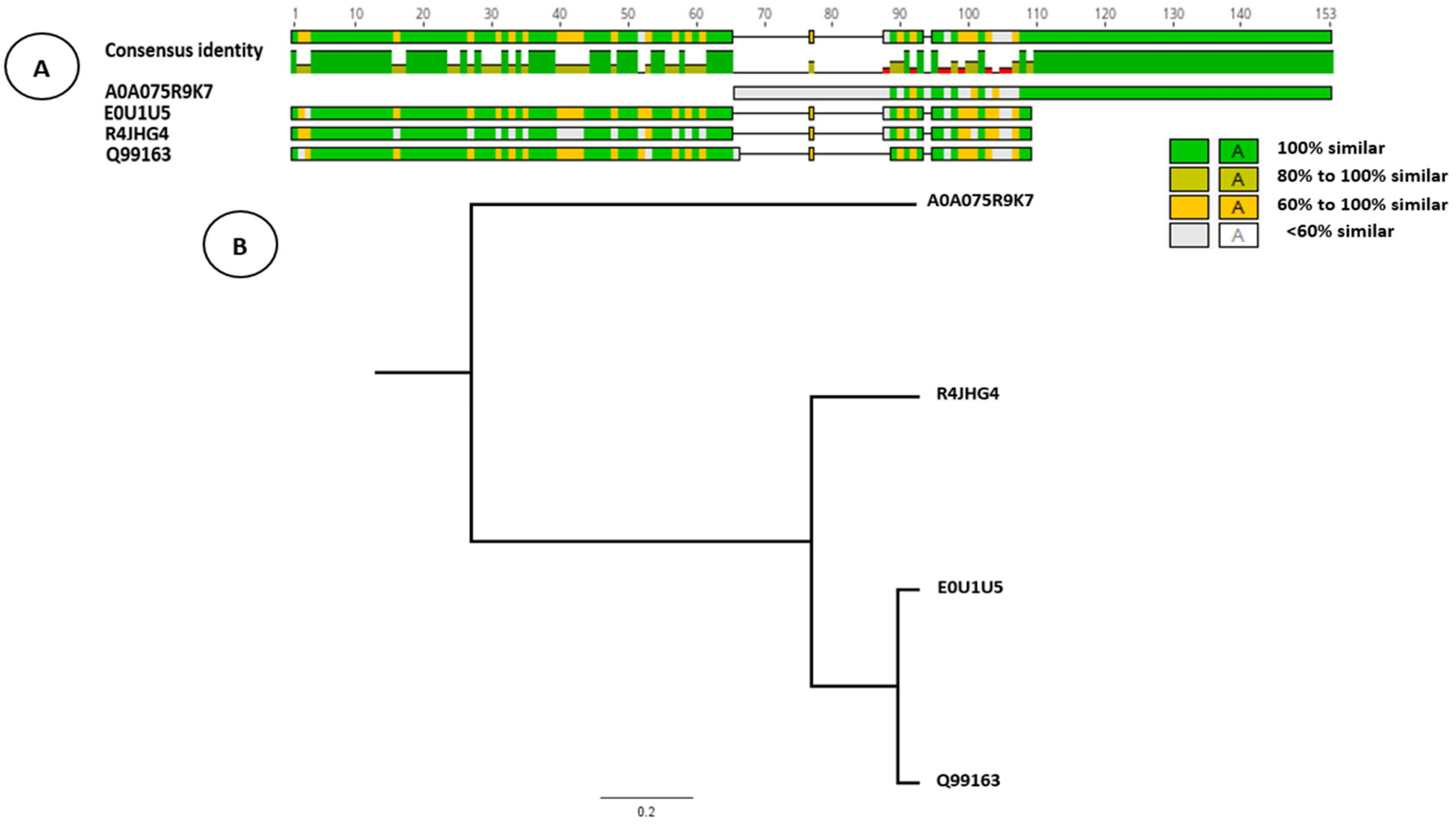

Figure S20. Amino acids alignment of N-acetylmuramoyl-L-alanine amidase protein (A0A518VEA4) encoded in PBSX-like region of *Bl* 1821L and Bl 1951 genome with the similar proteins of the defective prophages PBSZ, PBSX, and PBP180 using the programme CLUSTALO.

```
A0A518VEA4  A0A518VEA4_BRELA     1    -------------------MEKPILIIDAGHG----GADPGASGNHMQEKDLTLQIGLYQ    37
E0U1U7      E0U1U7_BACPZ         1    MVNIIQDFIPVGANNRPGYAMTPLYITVHNTANTAAGADAEAHARYLKNPDTATS-----    55
R4JMV0      R4JMV0_9CAUD         1    MVKIIQALIPKQNRNRPGNRMKPLYITVHNTSNTGRGADAANHAAFVARASTGVS-----    55
P39800      XLYA_BACSU           1    MVNIIQDFIPVGANNRPGYAMTPLYITVHNTANTAVGADAAAHARYLKNPDTTTS-----    55
                                                           .*: *   . .    ***    . .: . .   .

A0A518VEA4  A0A518VEA4_BRELA    38    LQRCRELNLPVAITRTTDTTLTPSQRTTLVKQSGATYCISNHINAGGGEGVEAIHSI--F    95
E0U1U7      E0U1U7_BACPZ        56    ------------WHFTVDD--------TEIYQHL--PLNENGWHAGDGNGSGNRASIGIE    93
R4JMV0      R4JMV0_9CAUD        56    ------------WHYTVDD--------QVIYQHL--PLNENGWHAGDGRGTGNMKSIGIE    93
P39800      XLYA_BACSU          56    ------------WHFTVDD--------TEIYQHL--PLNENGWHAGDGNGSGNRASIGIE    93
                                                     *.*           : *       .*  :**.*.*     **

A0A518VEA4  A0A518VEA4_BRELA    96    TSNKLANALAQAVAAEGQRFRRVYTRVGSDGRDYYFMHRETGAVETIIMEYGFIDHVGDA   155
E0U1U7      E0U1U7_BACPZ        94    ICENTDGDFAQATANAQWLIKTLMSEHN--------------ISLANVVPHKYWSGKECP   139
R4JMV0      R4JMV0_9CAUD        94    ICENAGANFEQAVKNAQWLIRKLMGDLG--------------IPLSNVVPHKHWSGKECP   139
P39800      XLYA_BACSU          94    ICENADGDFAKATANAQWLIKTLMAEHN--------------ISLANVVPHKYWSGKECP   139
                                       .::    : :*.      :: :    .                 : :: : . .

A0A518VEA4  A0A518VEA4_BRELA   156    QKLTNNWKRYAEAVLKAFSSHIGHPYSPALEEPIDDFQMAVDALVQA-------------   202
E0U1U7      E0U1U7_BACPZ       140    RKLLDTWDSFKAGIGGGGSQTYVVKQGDTLTSIARAFGVTVAQLREWNNIEDPNVIQVGQ   199
R4JMV0      R4JMV0_9CAUD       140    RKLLNRWDGFKAGIASASTSQMTTAKPVKETSI-------------------------KT   174
P39800      XLYA_BACSU         140    RKLLDTWDSFKAGIGGGGSQTYVVKQGDTLTSIARAFGVTVAQLQEWNNIEDPNLIRVGQ   199
                                      :** : *. :  .:  . :.           .

A0A518VEA4  A0A518VEA4_BRELA   203    ---------------------------------------KIITSPDYWKQNAGPNGTVLG   223
E0U1U7      E0U1U7_BACPZ       200    VLIVSAPSYAEEPELYPLPDGIIKLTTPYTSGEHVFQVQRALAALYFYPDKGAVNNGIDG   259
R4JMV0      R4JMV0_9CAUD       175    ISTRSTSKTNKVKKTYSLPAGILKVTKPLTKGSAVKAVQQALASIYFYPDKGAINNGIDG   234
P39800      XLYA_BACSU         200    VLIVSAPSAAEKPELYPLPDGIIQLTTPYTSGEHVFQVQRALAALYFYPDKGAVNNGIDG   259
                                                                             : :::  :: ::.. *. : *

A0A518VEA4  A0A518VEA4_BRELA   224    DYAAQLIKNMA-----NHLKAGA----------------                        241
E0U1U7      E0U1U7_BACPZ       260    IYGPKTADAVARFQSVNGLTADGIYGPATKAKIAAQLS-                        297
R4JMV0      R4JMV0_9CAUD       235    YYGPKTANAVSRFQMMHGLTPDGIYGPKTKETLSKVIES                        273
P39800      XLYA_BACSU         260    VYGPKTADAVARFQSVNGLTADGIYGPATKEKIAAQLS-                        297
                                       *. :  . ::     : *. ..
```

***Highlighted dark grey color amino acids denote similarity among the aligned N-acetylmuramoyl-L-alanine amidase proteins. (Refer to SM Figure S21 & Tables S19 and S20)**

Table S19. **Distance matrices of N-acetylmuramoyl-L-alanine amidase protein (A0A518VEA4) encoded in PBSX-like region of *Bl* 1821L and *Bl* 1951 with the similar proteins of the defective prophages PBSZ, PBSX, and PBP180 .**

Uniprot Accession # (https://www.uniprot.org)	Uniprot Accession # (https://www.uniprot.org)			
	**A0A518VEA4**	**E0U1U7**	**R4JMV0**	**P39800**
**A0A518VEA4**		0.22	1.75	1.40
**E0U1U7**	0.22		0.55	0.05
**R4JMV0**	1.75	0.55		0.54
**P39800**	1.40	0.05	0.54	

Table S20. **Alignment (%) of N-acetylmuramoyl-L-alanine amidase protein (A0A518VEA4) encoded in PBSX-like region of *Bl* 1821L and *Bl* 1951 with the similar proteins of the defective prophages PBSZ, PBSX, and PBP180.**

Uniprot Accession # (https://www.uniprot.org)	Uniprot Accession # (https://www.uniprot.org)			
	**A0A518VEA4**	**E0U1U7**	**R4JMV0**	**P39800**
**A0A518VEA4**		15.4	7.7	15.4
**E0U1U7**	15.4		53.5	95.3
**R4JMV0**	7.7	53.5		53.9
**P39800**	15.4	95.3	53.9	

Figure S21. Schematic presentation of amino acids alignment of N-acetylmuramoyl-L-alanine amidase protein (A0A518VEA4) encoded in PBSX-like region of *Bl* 1821L and *B*l 1951 with the similar proteins of the defective prophages PBSZ, PBSX, and PBP180 using programme Geneious basic (A) and CLUSTALO (B).

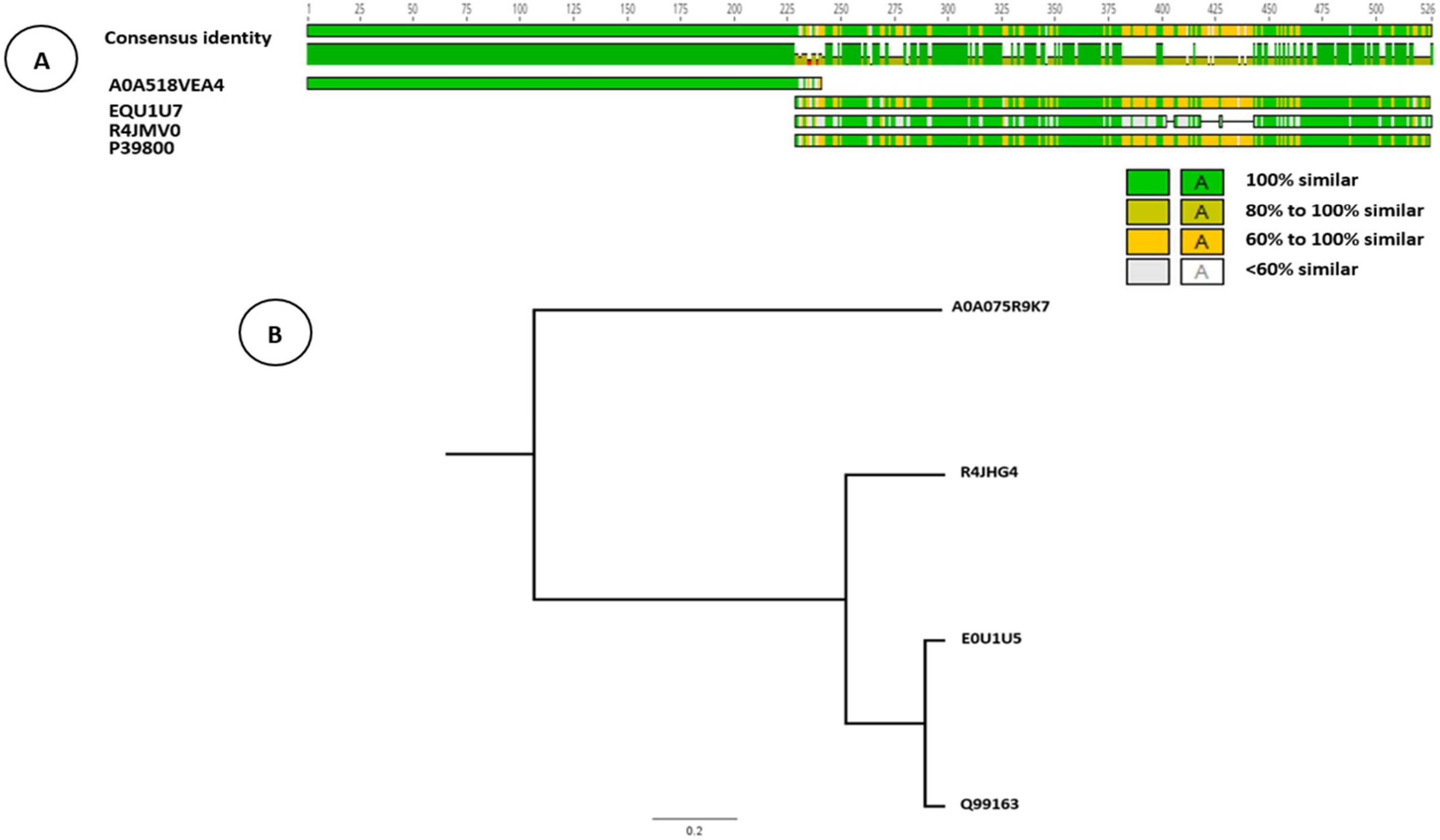

Supplement: Supplementary file 1 [file biomolecules-12-01154-s001.zip › biomolecules-1836609-supplementary.pdf]
